# Supplementary material for: Genomic Engineering of Oral Keratinocytes to Establish In Vitro Oral Potentially Malignant Disease Models as a Platform for Treatment Investigation
Source: Cells. 2024 Apr 19;13(8):710. doi: 10.3390/cells13080710 (PMC11049138; doi:10.3390/cells13080710)
Supplement: Supplementary file 1 [file cells-13-00710-s001.zip › Cells_Supplementary_Figure_Legends_R1.pdf]

Supplementary Figure S1: Growth curves, mutations and CNA plots for all individual cell cultures. For each patient biopsy the CNA plot is provided for the parental culture and all other sequenced genomically engineered cultures. In addition, the mutations obtained through targeted mutational sequencing are shown with corresponding variant allele frequencies (VAFs) and adjacent gene names and protein changes when applicable. Mutations depicted in red represent mutations that were introduced by genomic engineering using CRISPR/Cas9. All other mutations identified are shown in green. On the top right the number of population doublings at which the culture was sequenced, is indicated. For each patient biopsy the respective growth curves of the cell cultures are combined in the bottom right figure. This figure also shows at what timepoint TERT was introduced to the parental culture (gold arrow) or to the genomically engineered cultures (dark red arrow). The colored squares indicate at which timepoint and population doubling each modified cell line was sequenced and therefore corresponds to the presented CNA and mutation plots. All colors that indicate genomic modifications correspond to the same colors as used in Figures 1, 2 and 3.

Supplementary Figure S2: Per base coverage of the *CDKN2A* gene for each culture included in the sequencing.

Supplementary Figure S3: Per base coverage of the *TP53* gene for each culture included in the sequencing.

Supplementary Figure S4: Western blot for expression of p16 for a panel of cell lines.

Supplementary Figure S5: Western blot for expression of p53 for a panel of cell lines.

Supplementary Figure S6: Frequency of genetic changes in growing compared to non-growing oral leukoplakia cell cultures. Frequency of growing lines (blue) and non-growing lines (red) are shown as percentage of the total number of cultures for each category. On the right the p-valued determined by the Fisher's exact test for each genetic change is shown.

Supplementary Figure S7: CNAs and mutations for three cell cultures at different population doublings. For each culture the CNA plot is provided at two to four different population doublings. In addition, the mutations obtained through targeted mutational sequencing are shown with corresponding VAFs and adjacent gene names and protein changes if applicable. Red mutations represent mutations that were introduced by genomic engineering using CRISPR/Cas9. All other mutations are shown in green. On the top right the number of population doublings at which the culture was sequenced is indicated.

Supplementary Figure S8: Effectivity of PLK1 inhibitor GSK461364 for OL treatment. **Top:** Dose-response curves showing the relative cell viability of modified cell lines (black) with sensitive tumor line UM-SCC-22A (red) and epithelial line UPPP60 (green) as a reference indicating the therapeutic window of PLK1 inhibitor GSK461364. Experiments were performed three times in triplicate and the averaged value of the 3 experiments is presented. **Bottom:** Plot showing the IC50 for PLK1 inhibitor GSK461364 in all included cell lines. Samples are sorted based on cell type as indicated by color. The dotted red line indicates the IC50 for UM-SCC-22A, defined as the sensitive cell line.

Supplementary Figure S9: Effectivity of CHEK inhibitor LY2606368 for OL treatment. **Top:** Dose-response curves showing the relative cell viability of modified cell lines (black) with sensitive tumor line UM-SCC-22A (red) and epithelial line UPPP60 (green) as a reference indicating the therapeutic window of CHEK inhibitor LY2606368. Experiments were performed three times in triplicate and the averaged value of the 3 experiments is presented. **Bottom:** Plot showing the IC50 for CHEK inhibitor LY2606368 in all included cell lines. Samples are sorted based on cell type as indicated by color. The

dotted red line indicates the IC50 for UM-SCC-22A, defined as the sensitive cell line. The dotted green line indicates the IC50 for UPPP60, defined as the insensitive cell line.

Supplementary Figure S10: Effectivity of WEE1 inhibitor MK1775 for OL treatment. **Top:** Dose-response curves showing the relative cell viability of modified cell lines (black) with sensitive tumor line UM-SCC-22A (red) and epithelial line UPPP60 (green) as a reference indicating the therapeutic window of WEE1 inhibitor MK1775. Experiments were performed three times in triplicate and the averaged value of the 3 experiments is presented. **Bottom:** Plot showing the IC50 for WEE1 inhibitor MK1775 in all included cell lines. Samples are sorted based on cell type as indicated by color. The dotted red line indicates the IC50 for UM-SCC-22A, defined as the sensitive cell line. The dotted green line indicates the IC50 for UPPP60, defined as the insensitive cell line.
