# Supplementary material for: Genomic Engineering of Oral Keratinocytes to Establish In Vitro Oral Potentially Malignant Disease Models as a Platform for Treatment Investigation
Source: Cells. 2024 Apr 19;13(8):710. doi: 10.3390/cells13080710 (PMC11049138; doi:10.3390/cells13080710)
Supplement: Supplementary file 1 [file cells-13-00710-s001.zip › Supplementary_Table_S2.pdf]

Supplementary Table S2 Used medium, number of plated cells and incubation times for cell lines cultured for investigation of small molecule inhibitor experiments. In the cell line names, ^{KO} indicates that a knockout was performed for the specified gene, while $^{+}$ indicates that the gene was ectopically expressed.

Cell line	Medium	Cells per well for dose response analysis	CellTiter-Blue incubation time (minutes)
VU-preSCC-LP084-TERT^{+}	KGM	3000	180
VU-preSCC-LP121-p16^{KO}-TERT^{+}	KGM	3000	180
VU-preSCC-LP140-p16^{KO}-TERT^{+}	KGM	3000	180
VU-preSCC-HN1028-p16^{KO}-TERT^{+}	KGM	3000	180
VU-preSCC-M3	KGM	3000	180
VU-preSCC-M3-TERT^{+}	KGM	3000	180
UPPP-60	KGM	3000	180
UPPP-60-p16^{KO}-TERT^{+}	KGM	3000	180
UM-SCC-22A	DMEM	4000	120
