# Supplementary figures and images for: Genomic Engineering of Oral Keratinocytes to Establish In Vitro Oral Potentially Malignant Disease Models as a Platform for Treatment Investigation
Source: Cells. 2024 Apr 19;13(8):710. doi: 10.3390/cells13080710 (PMC11049138; doi:10.3390/cells13080710)

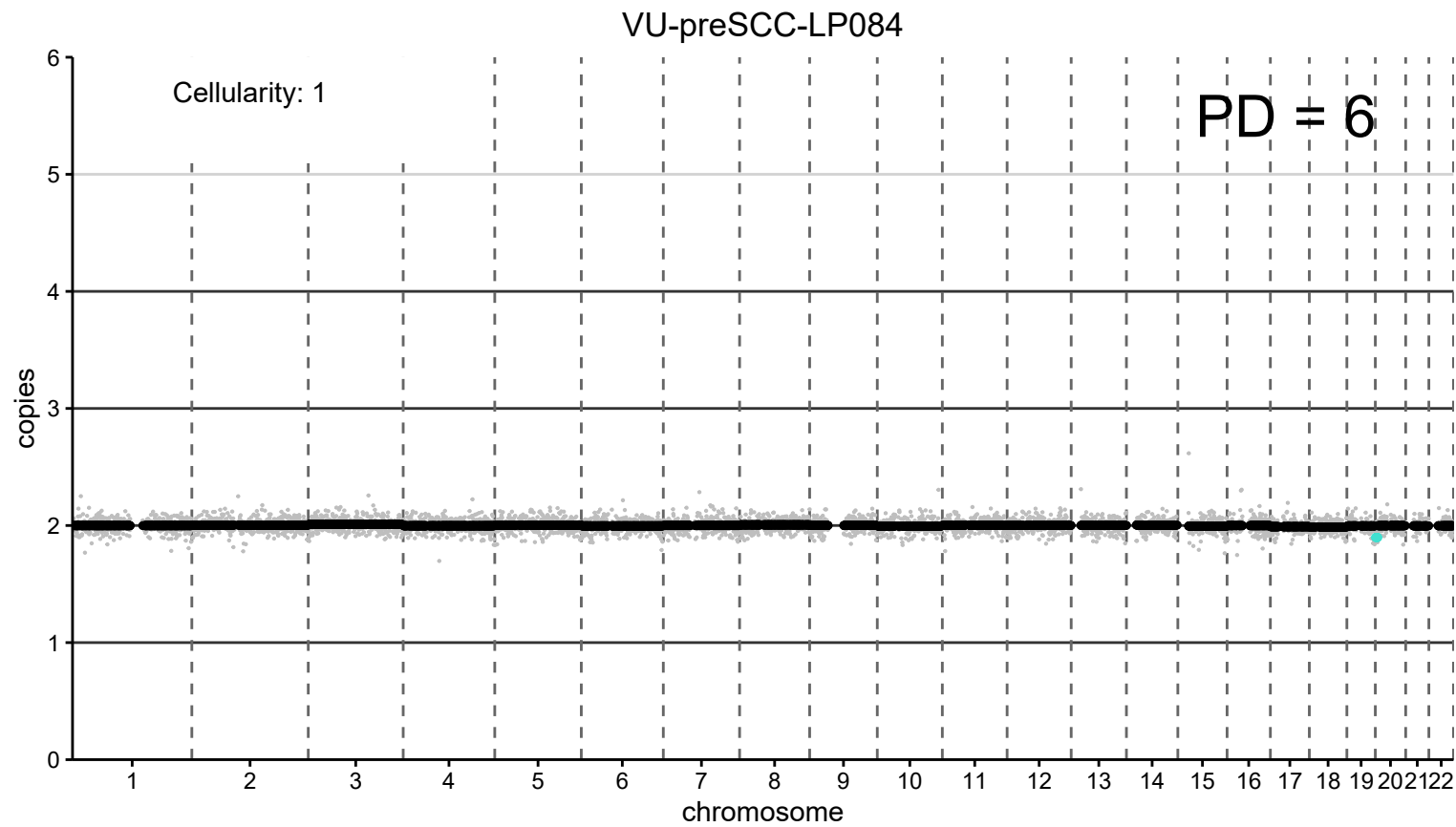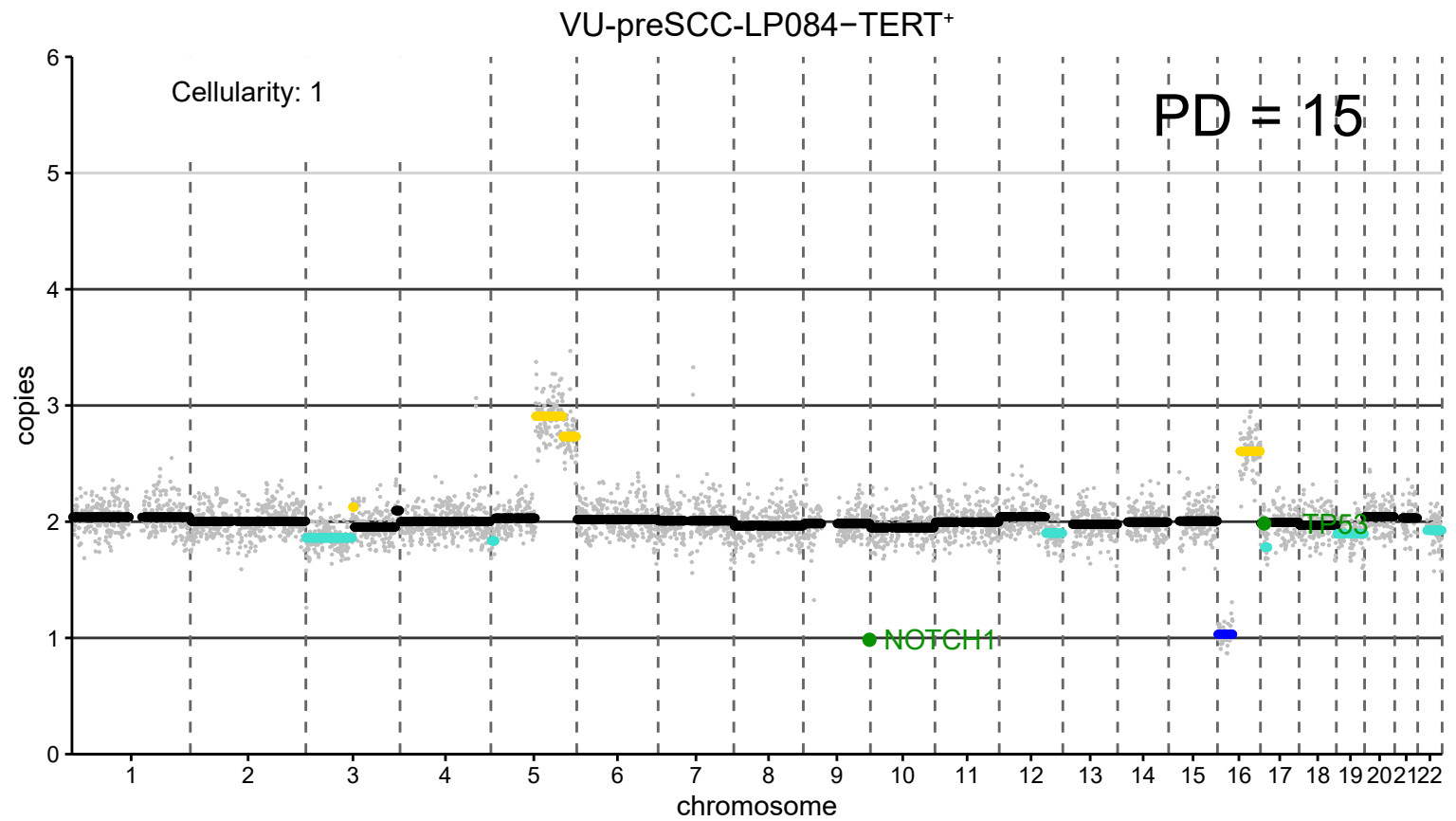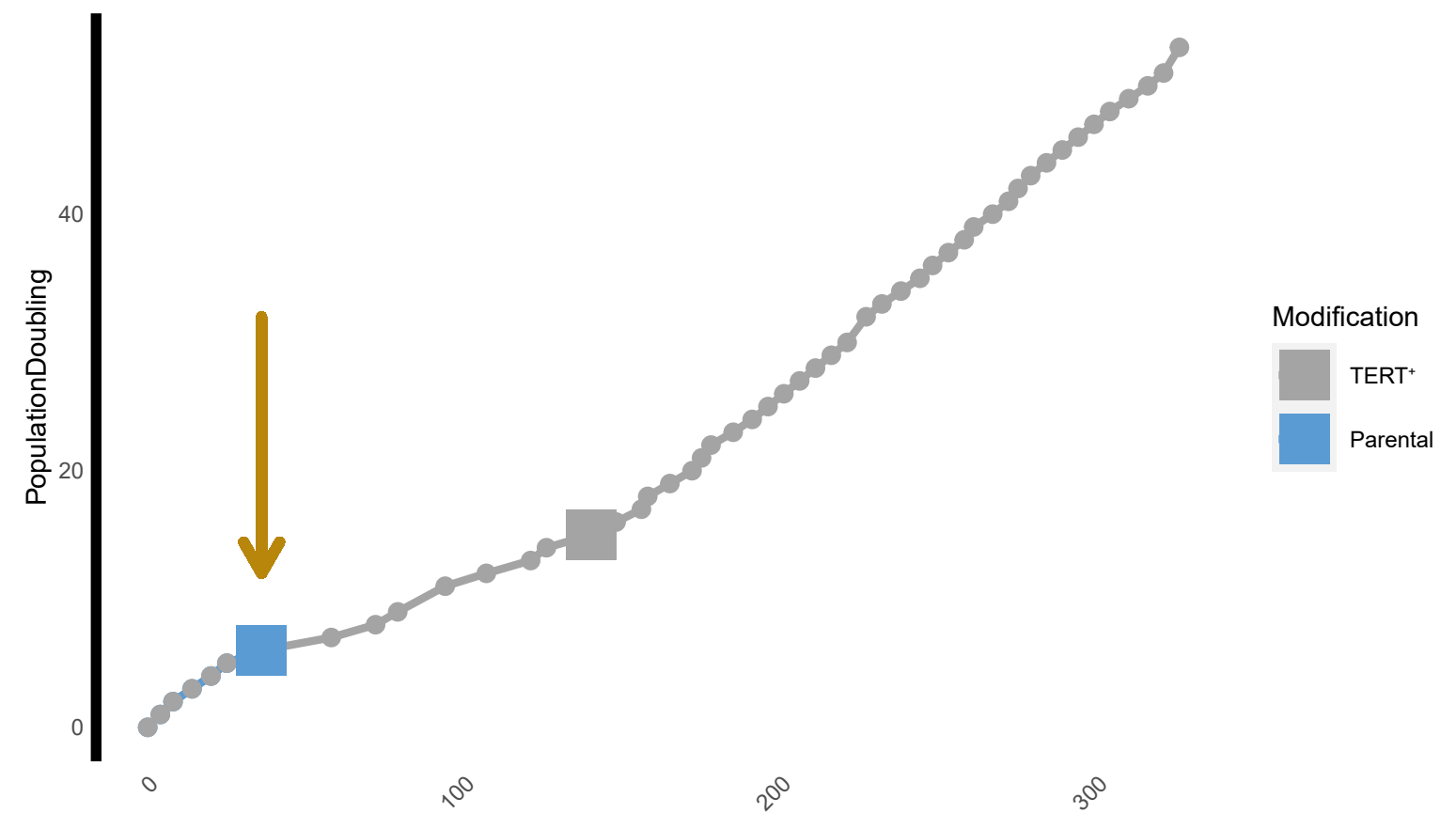

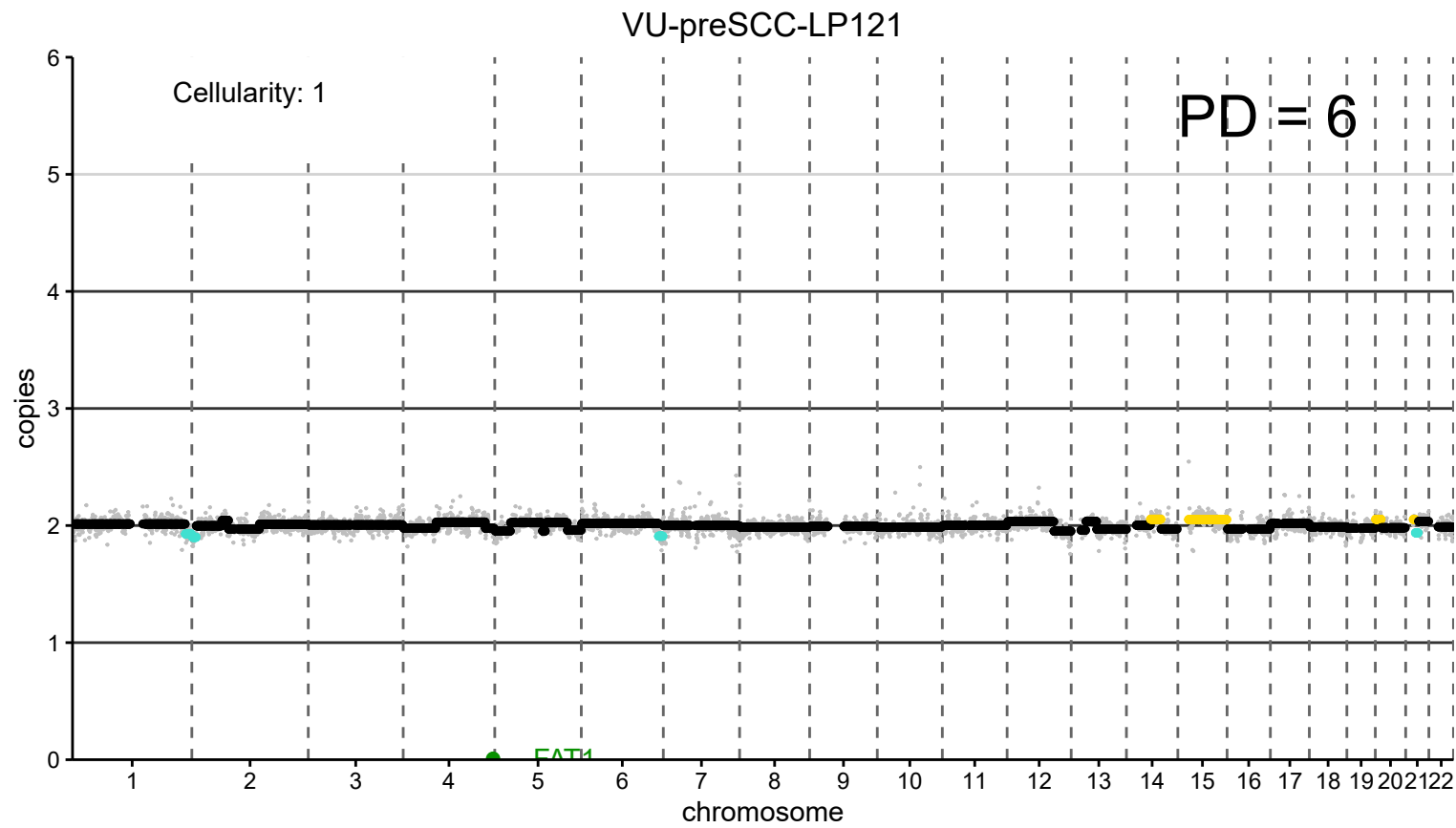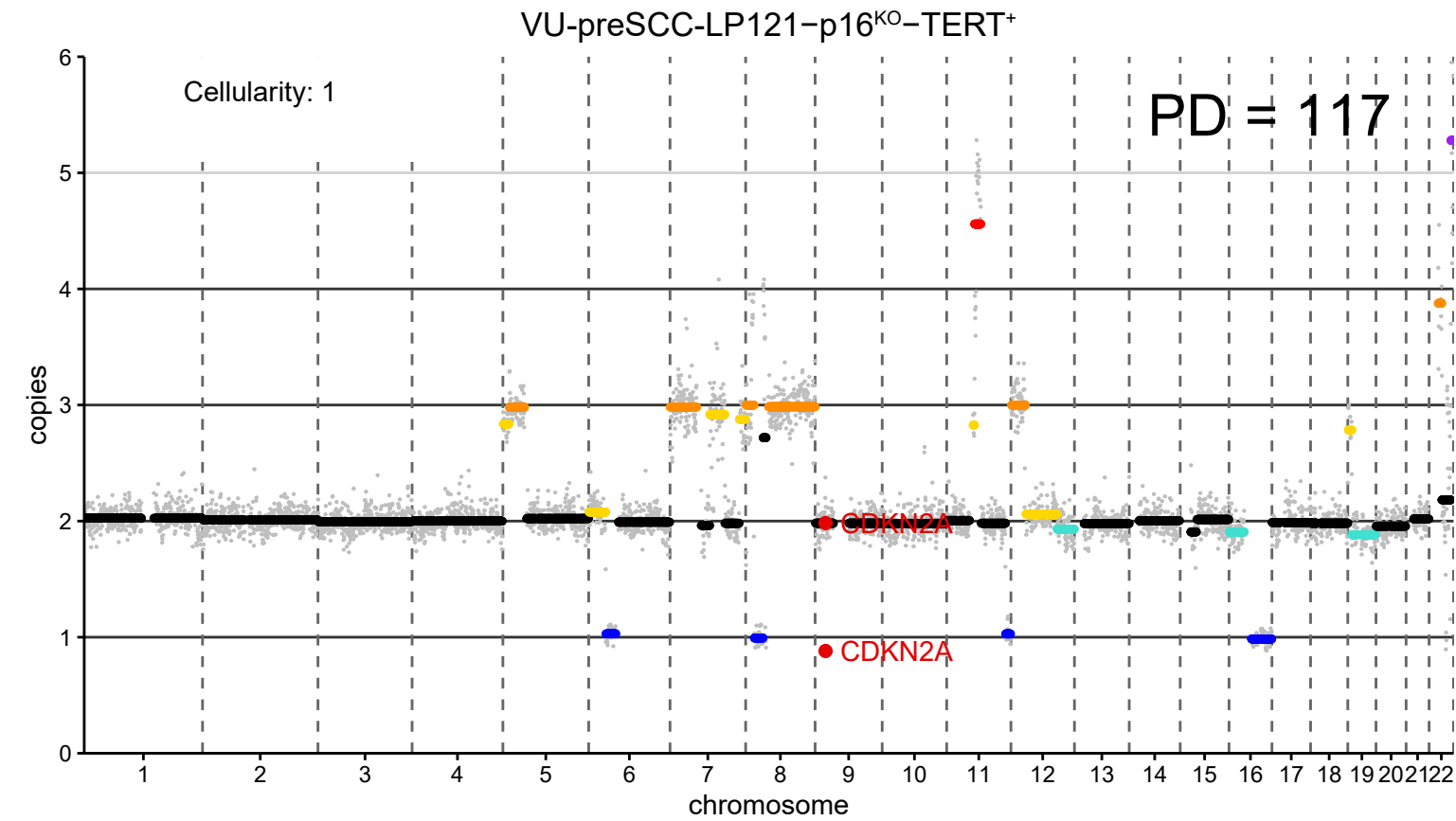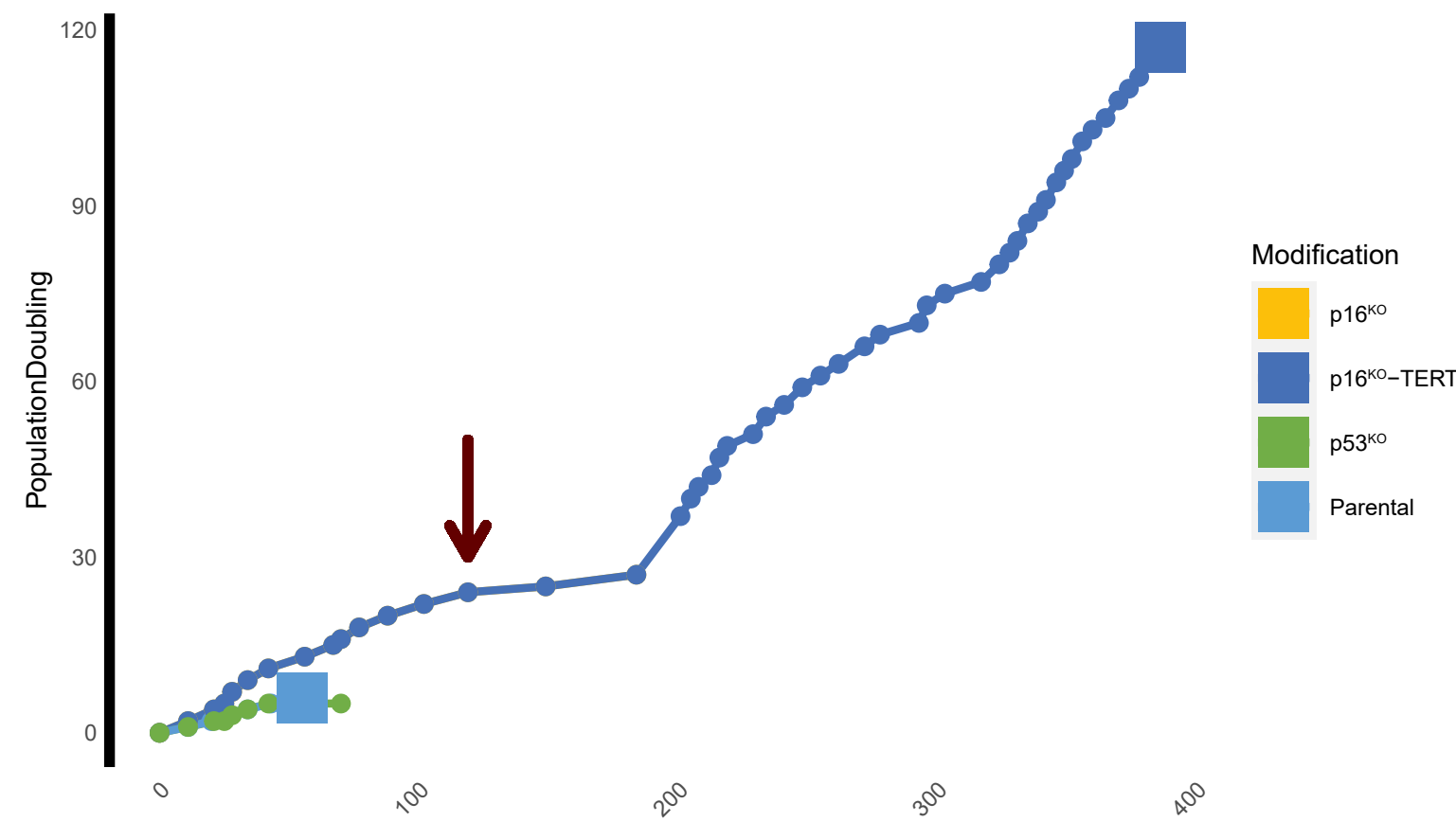

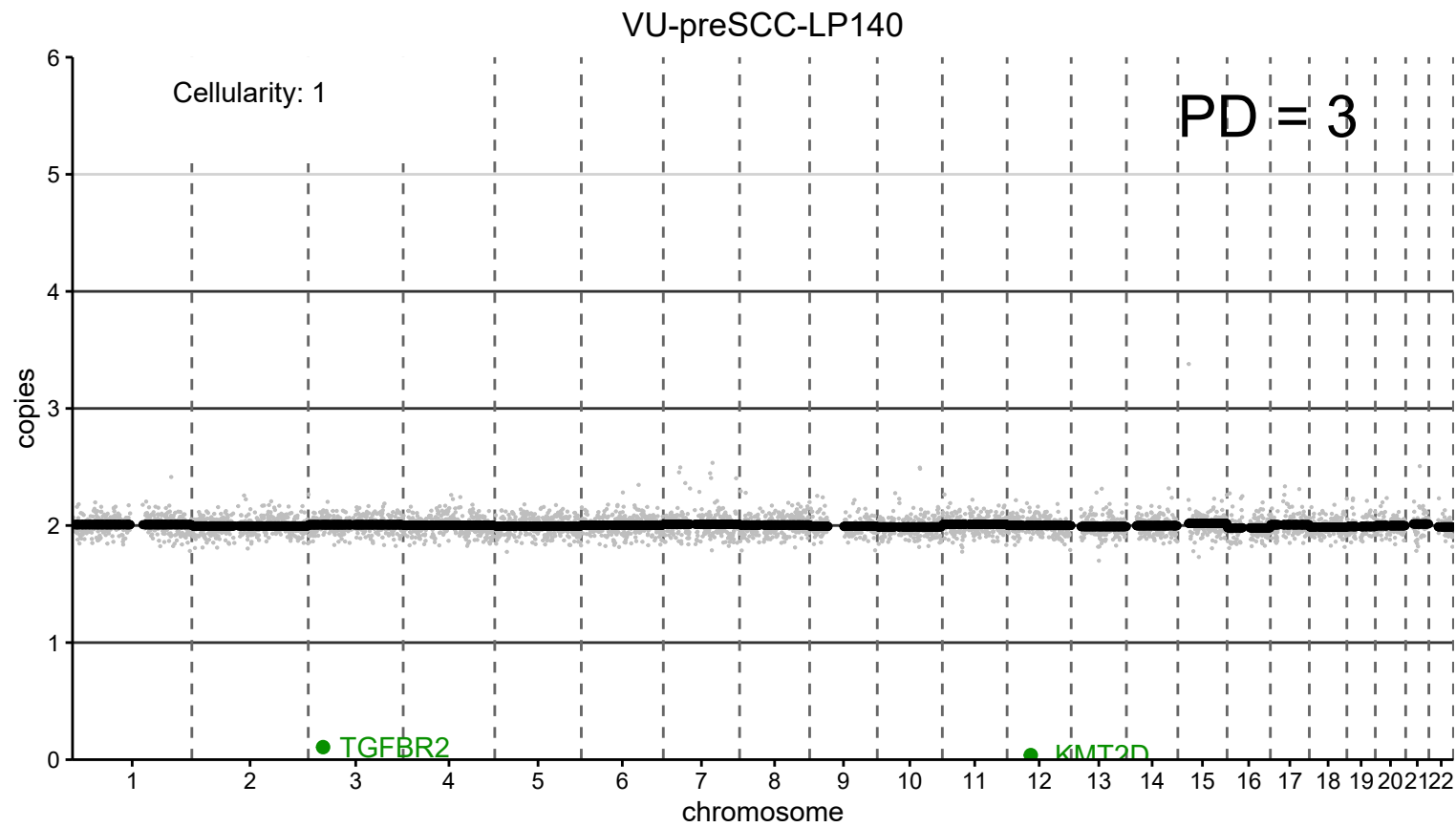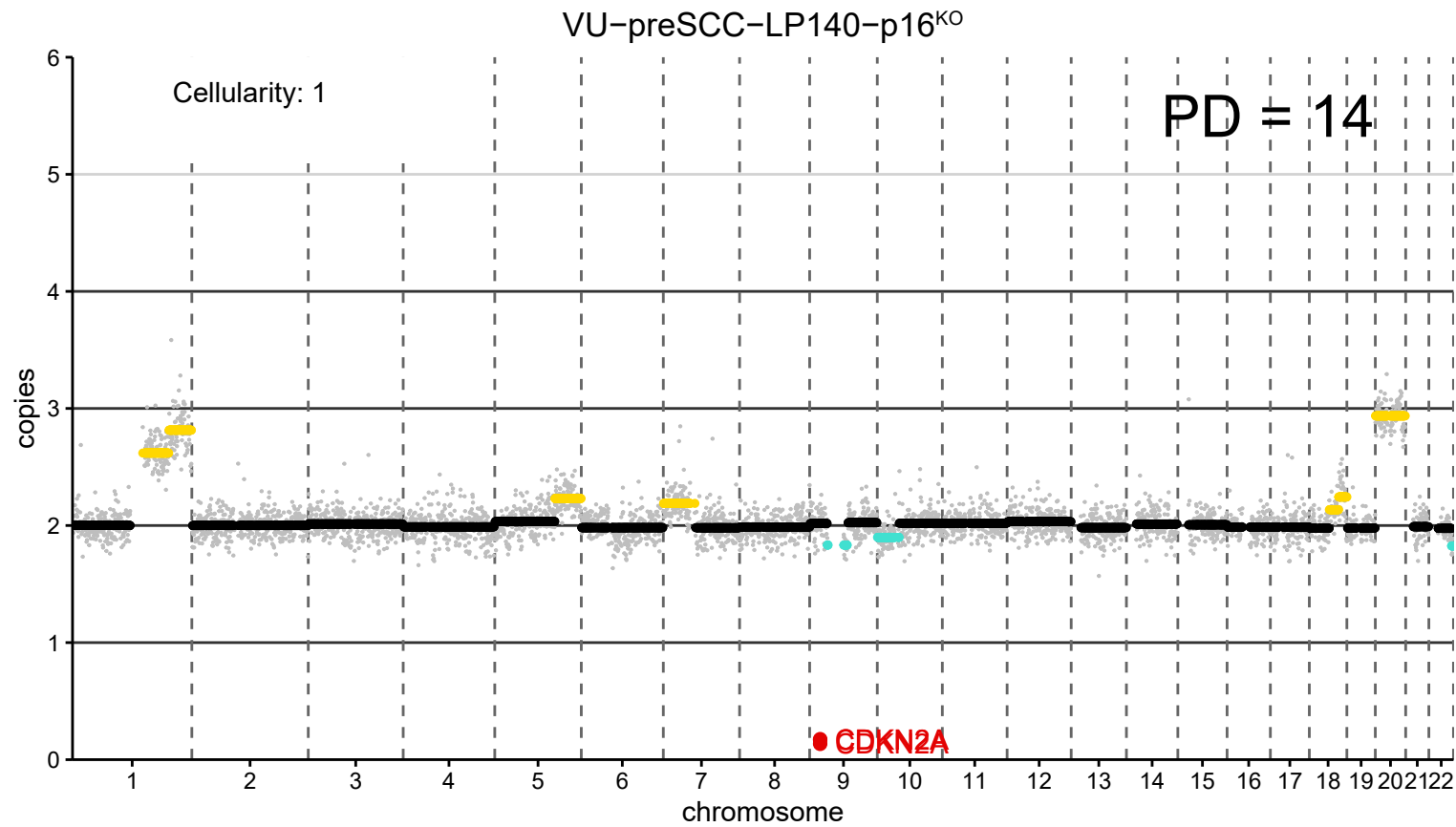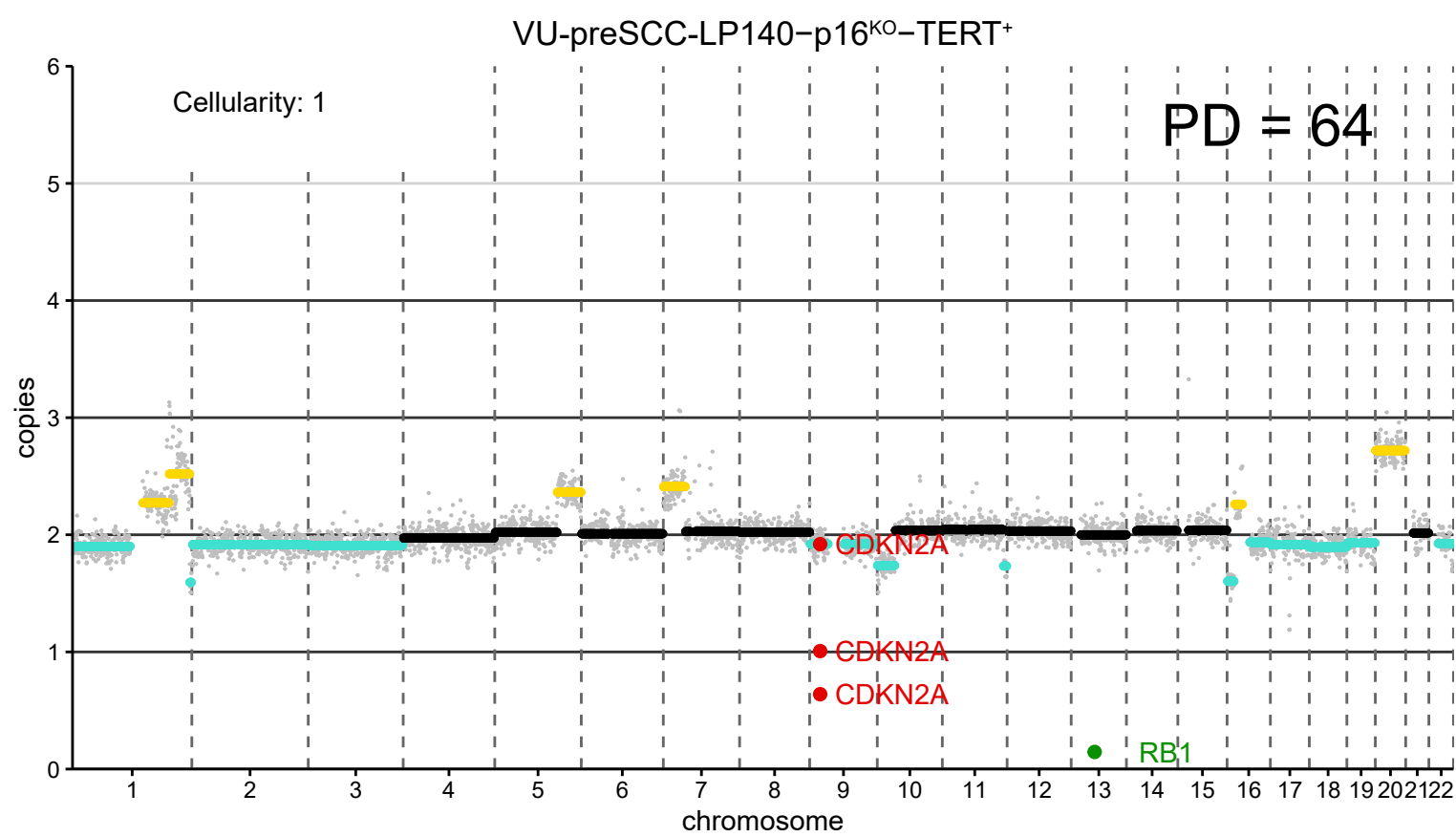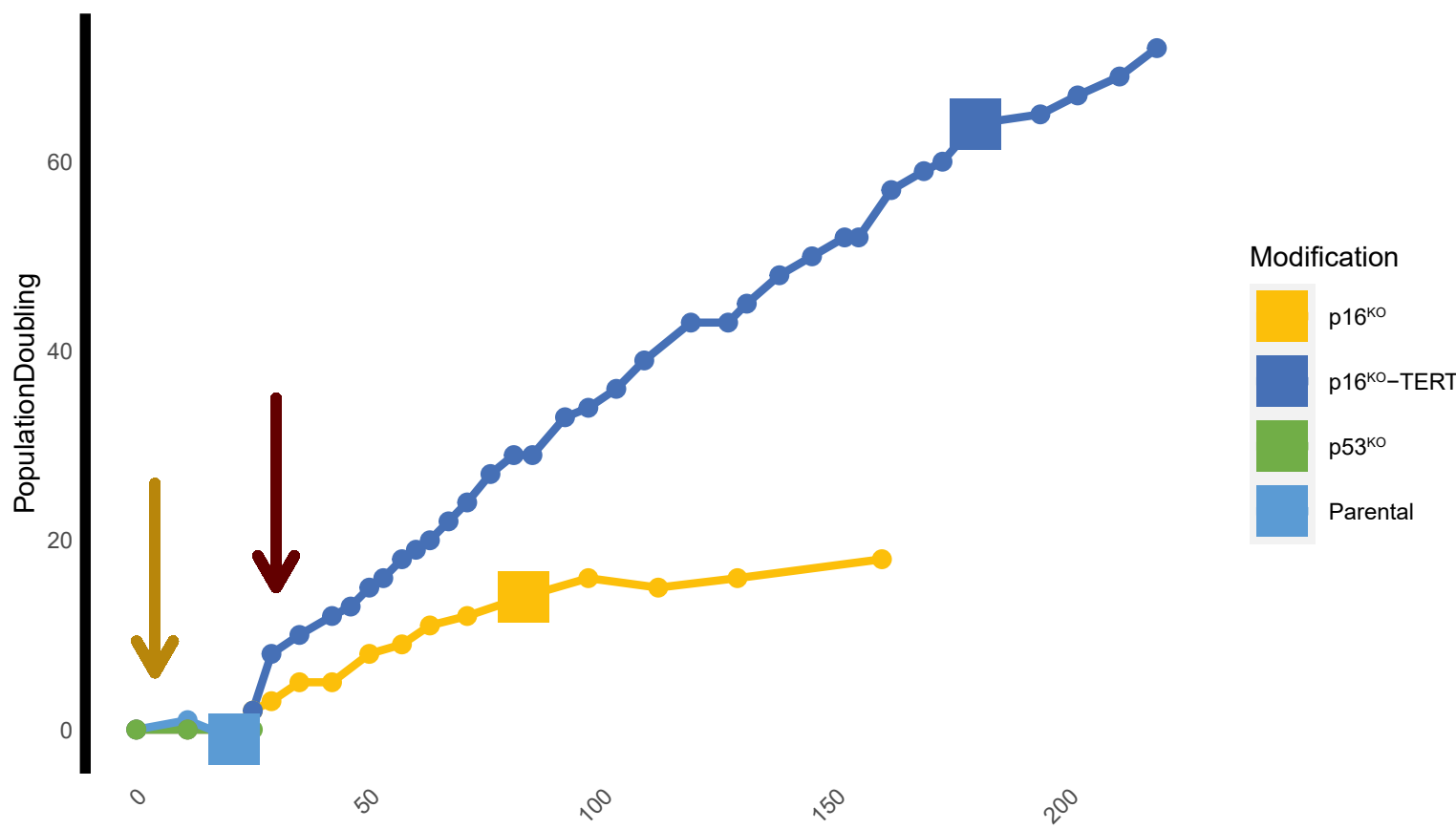

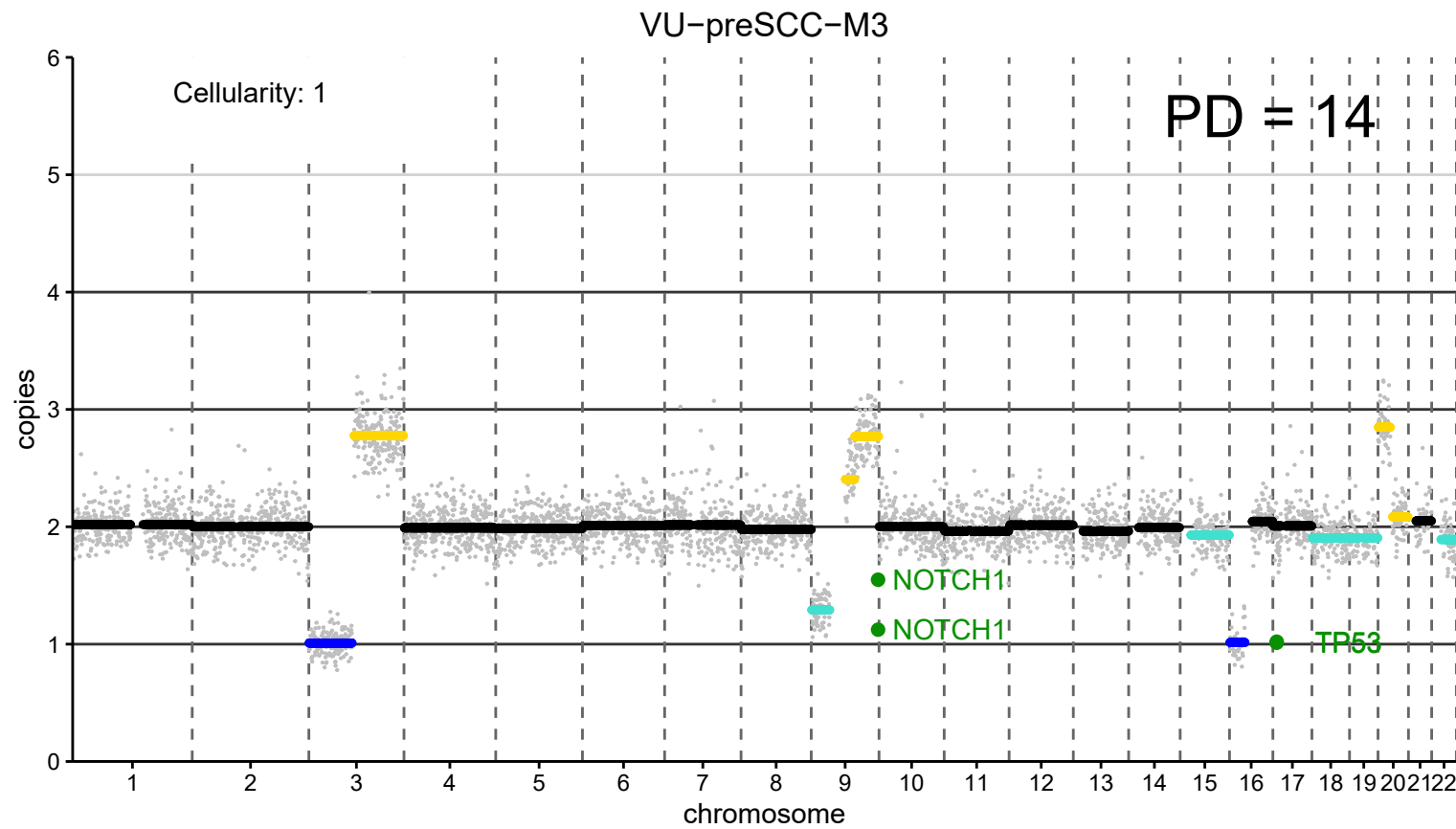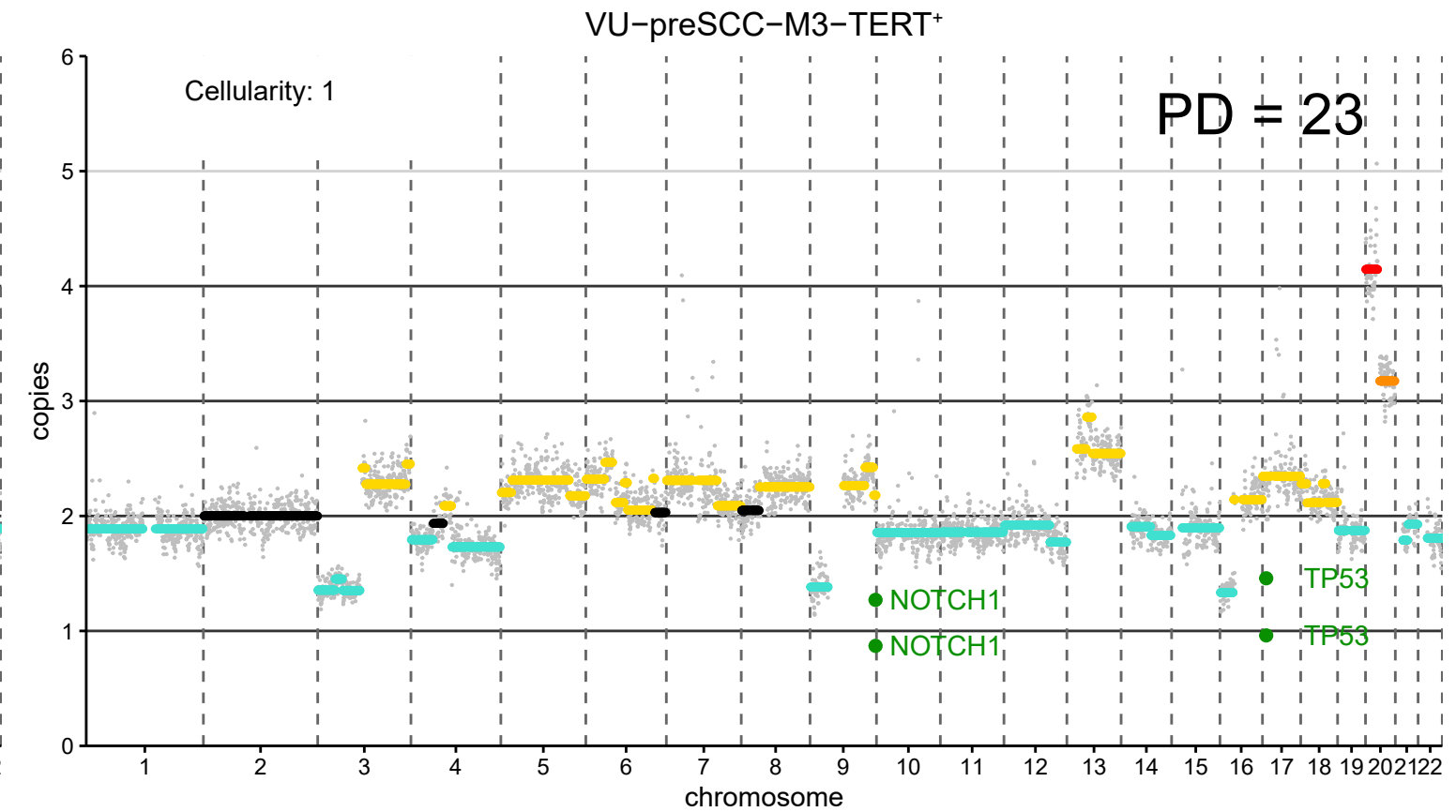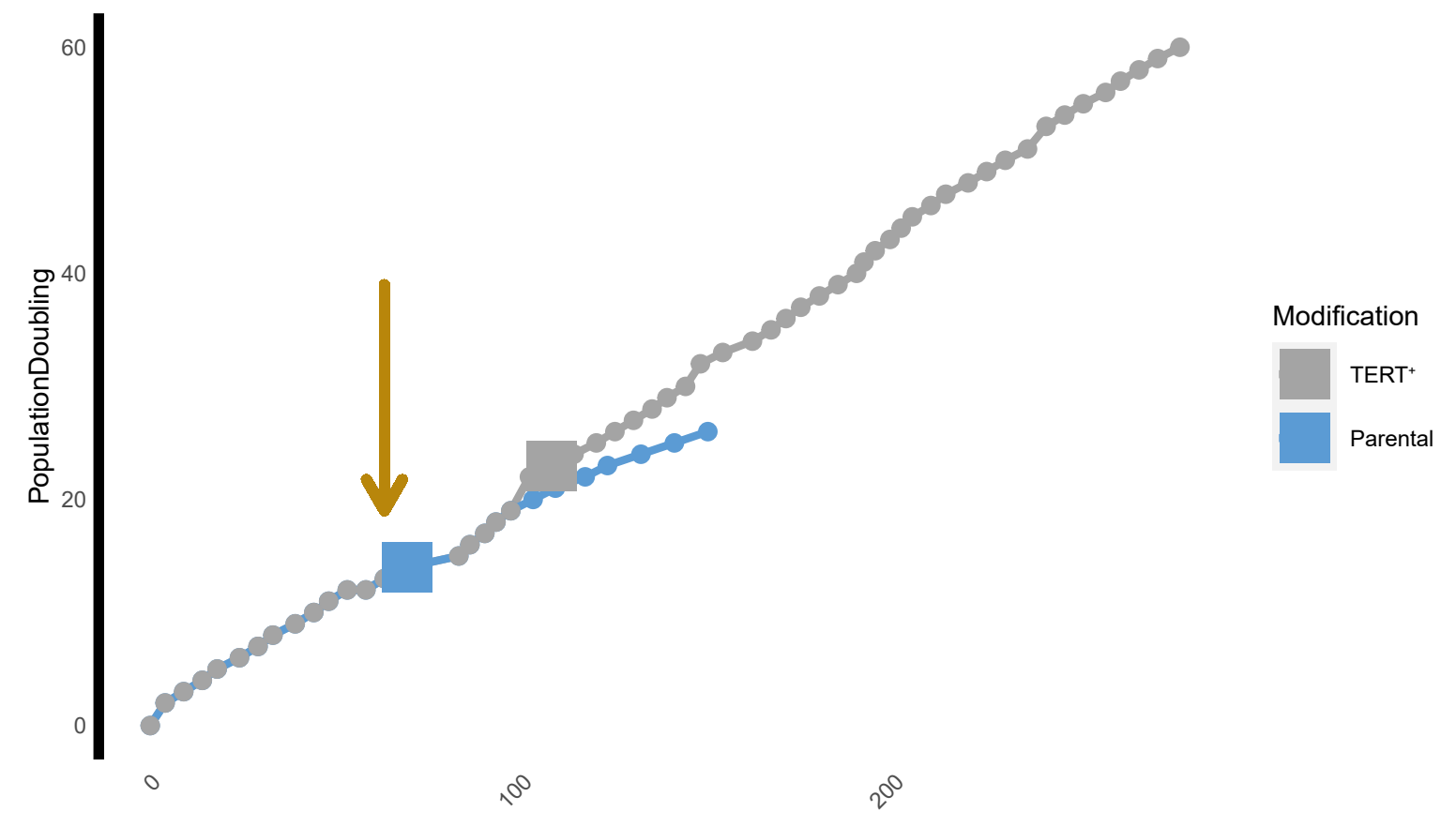

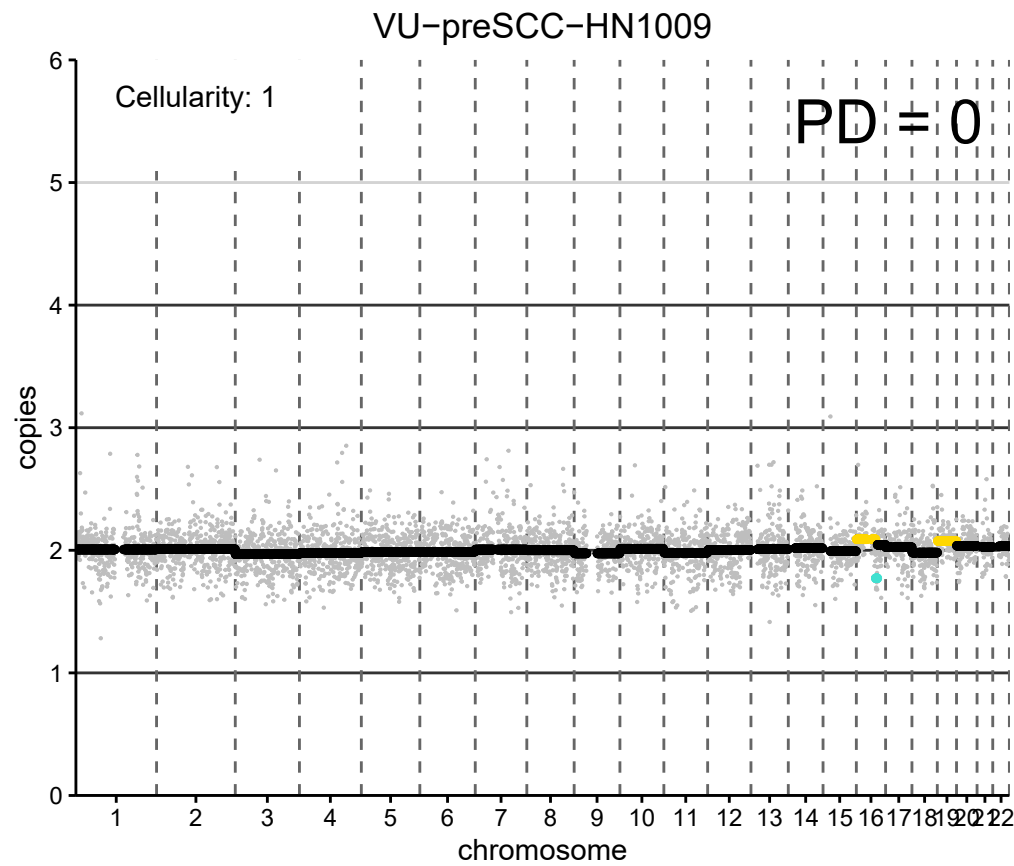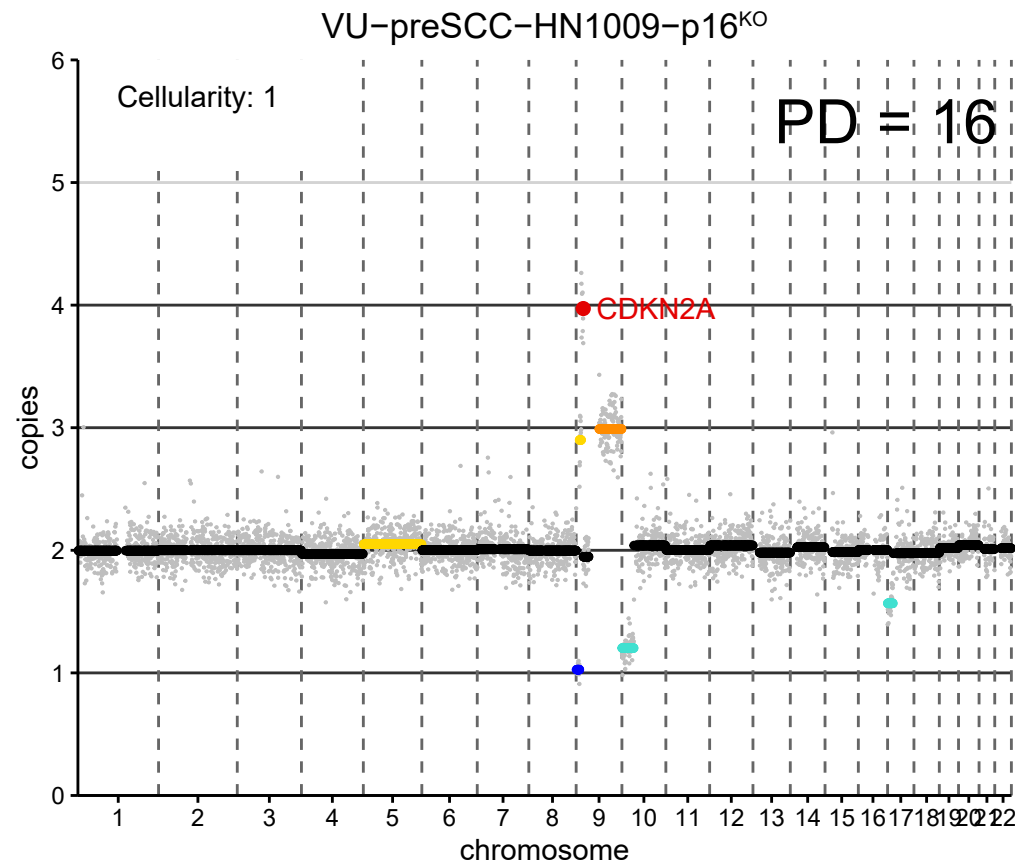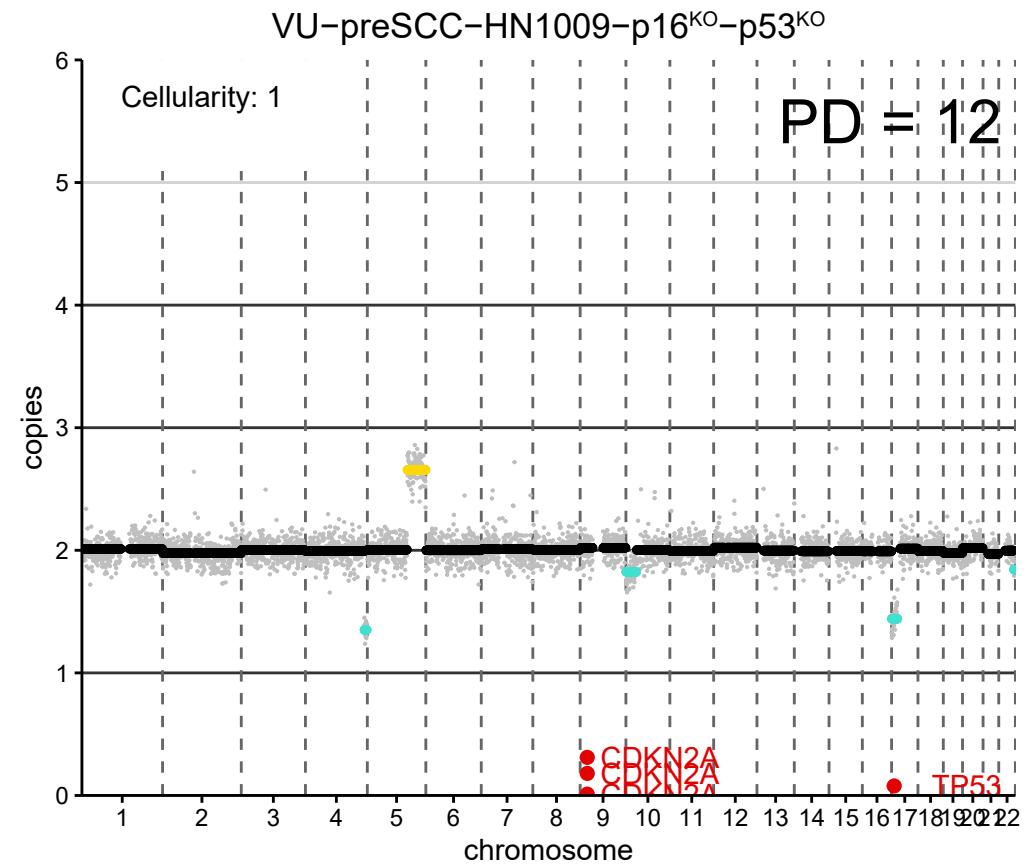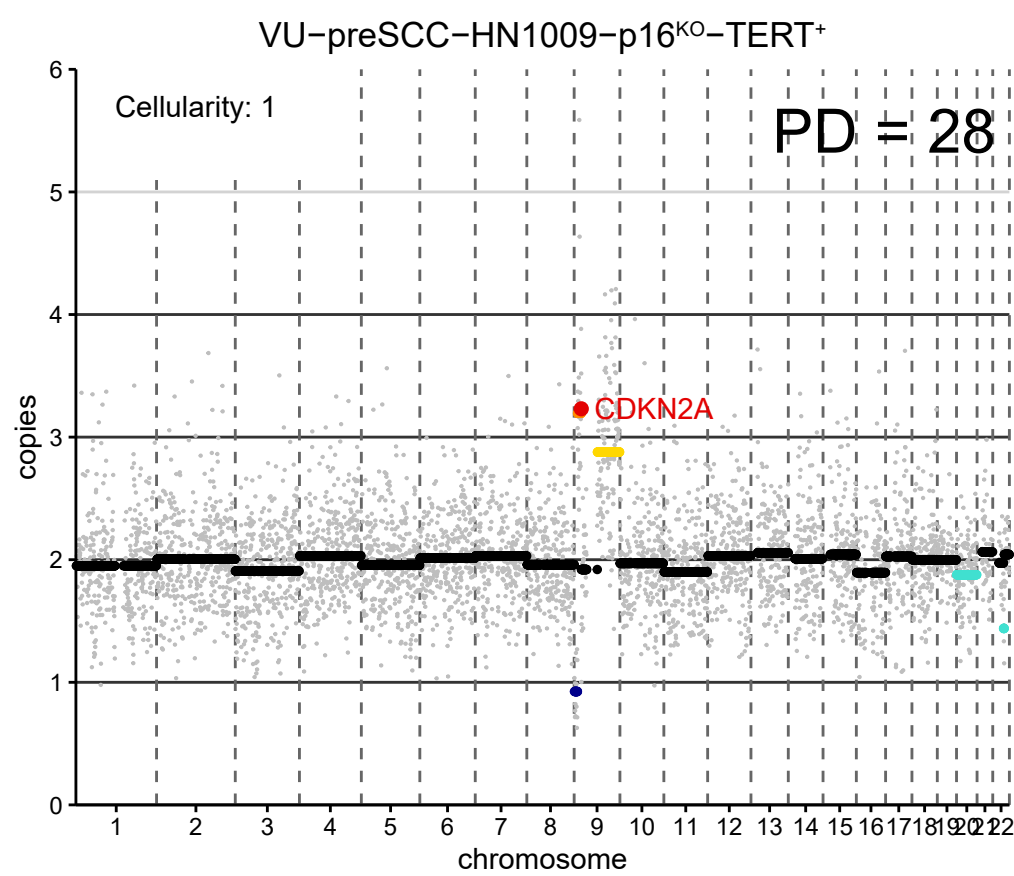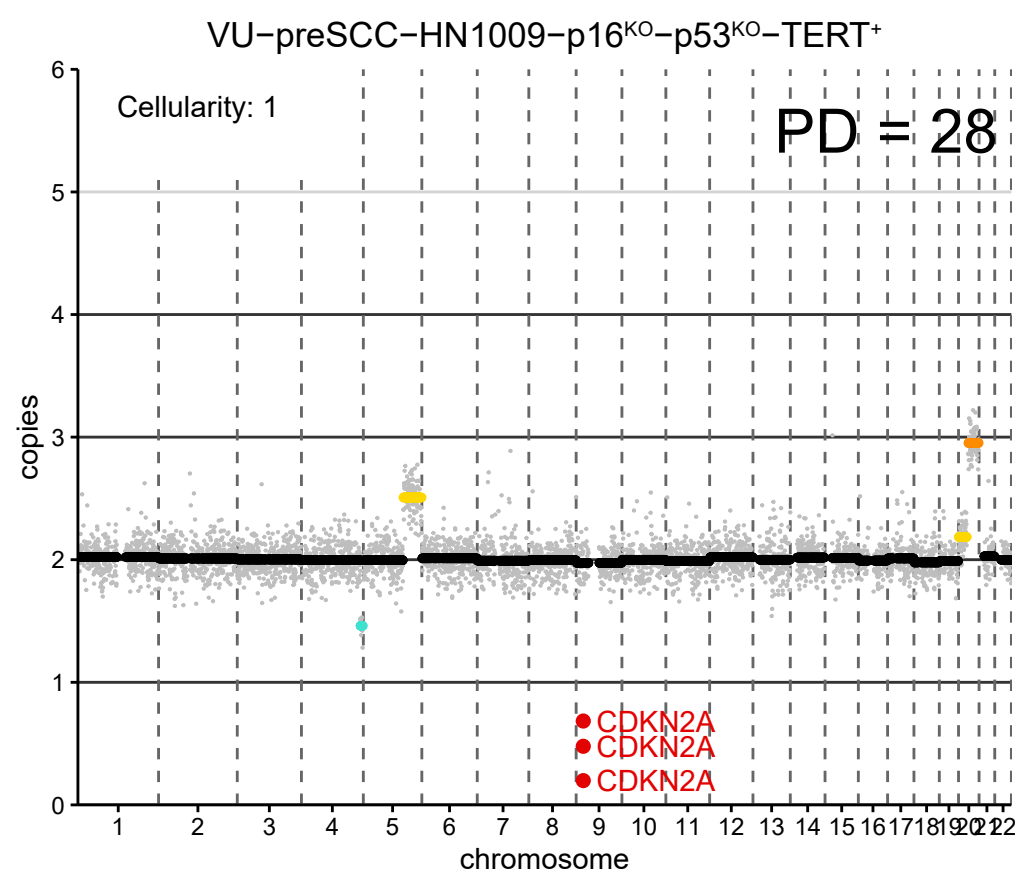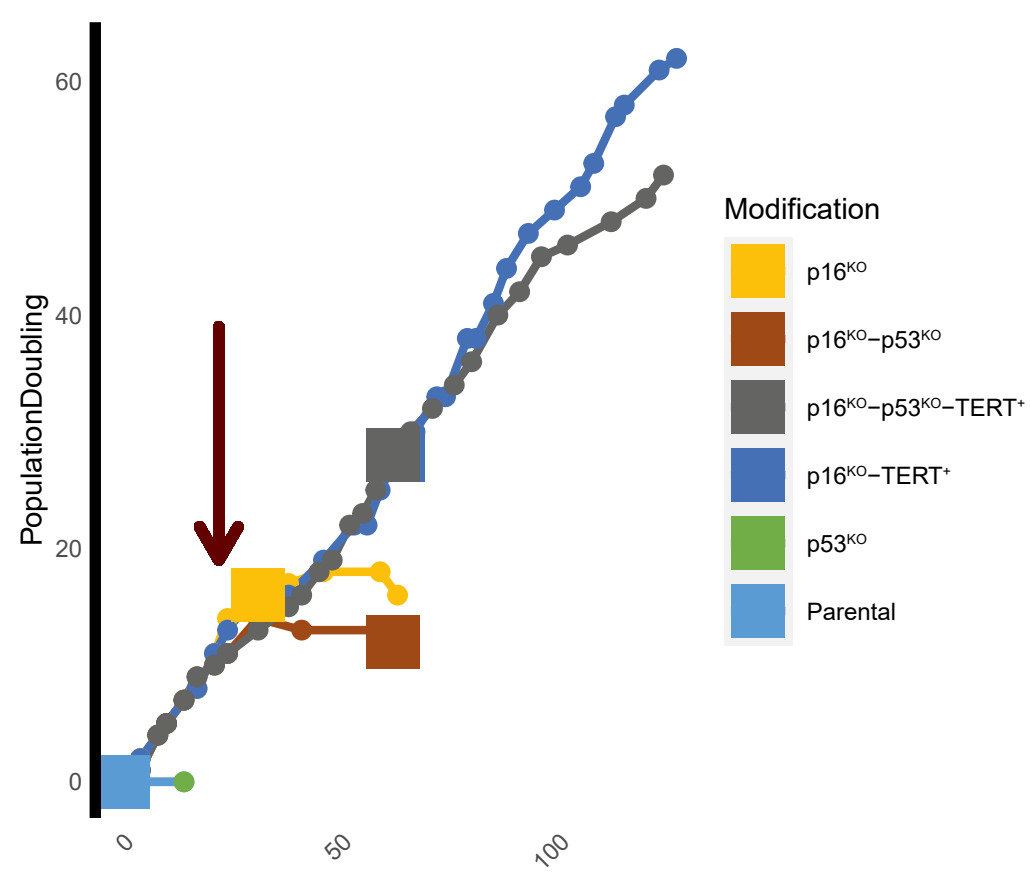

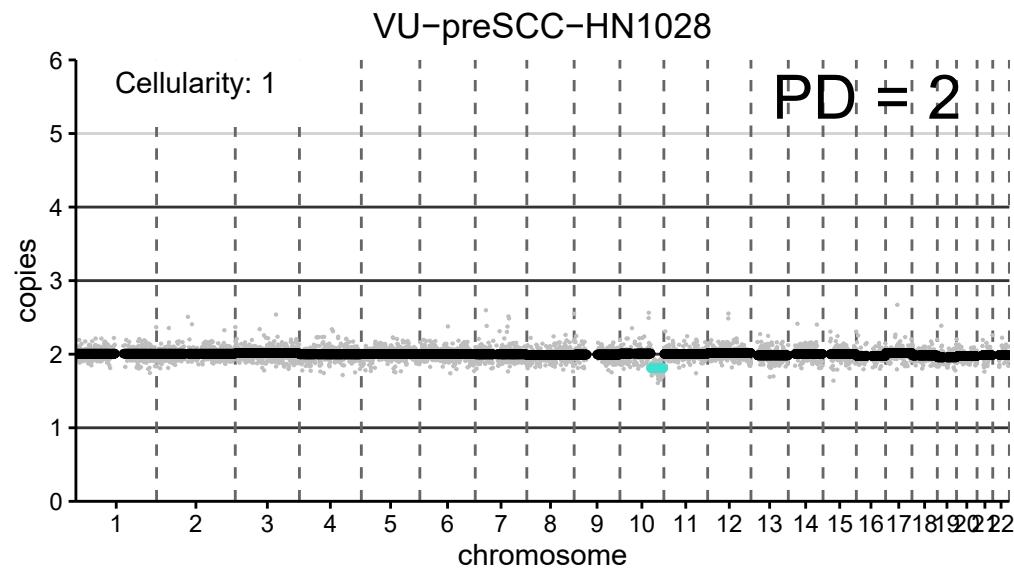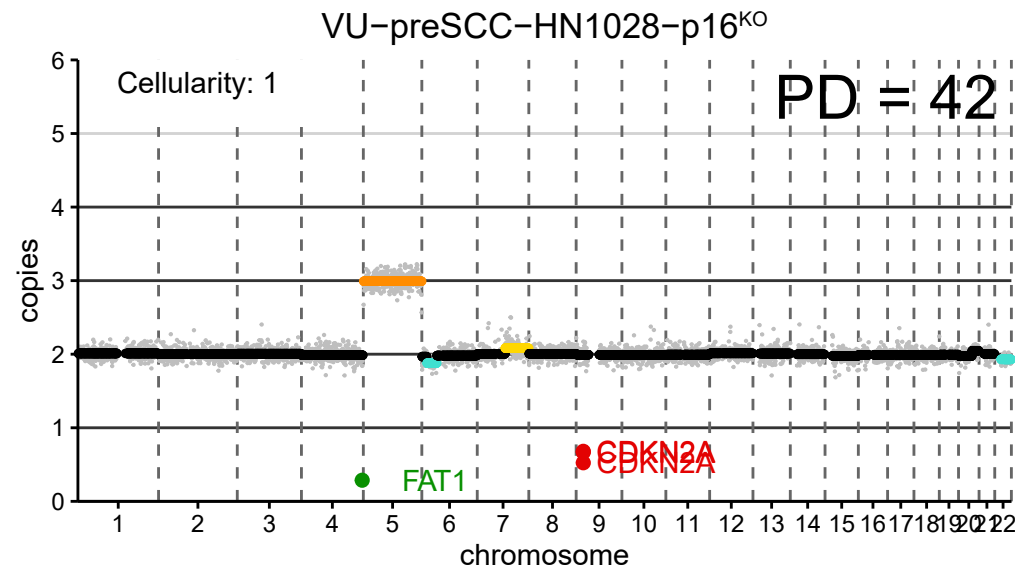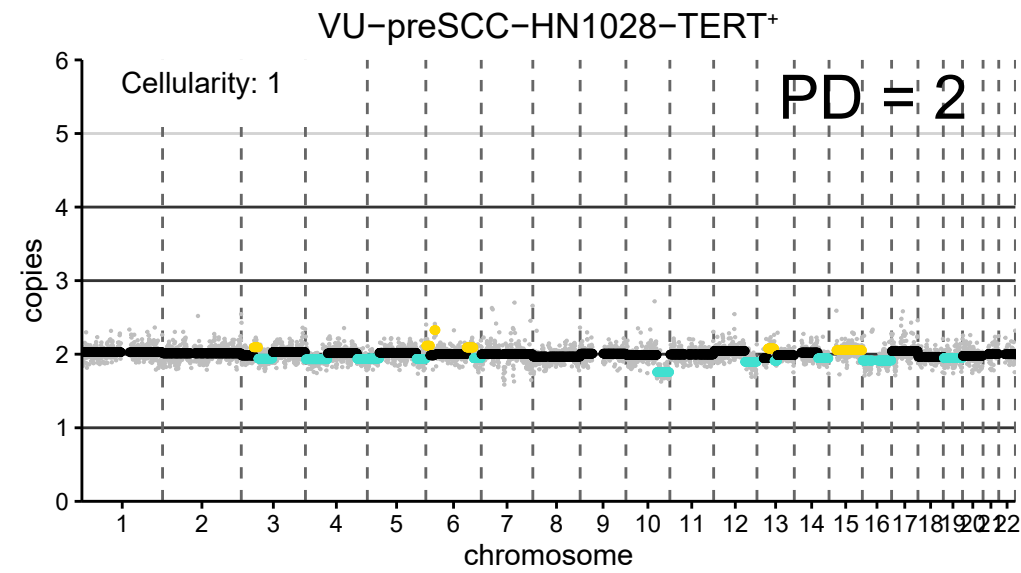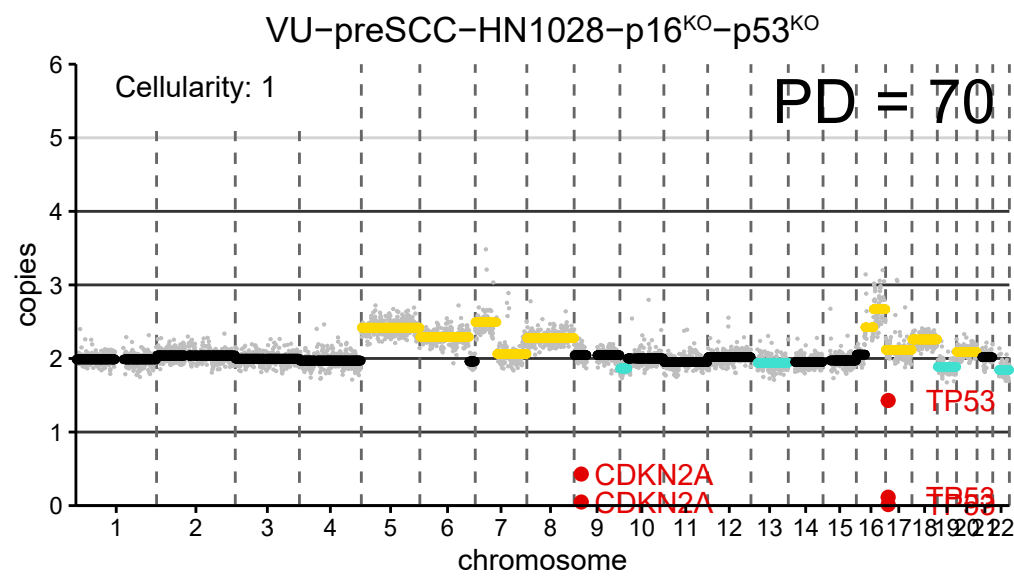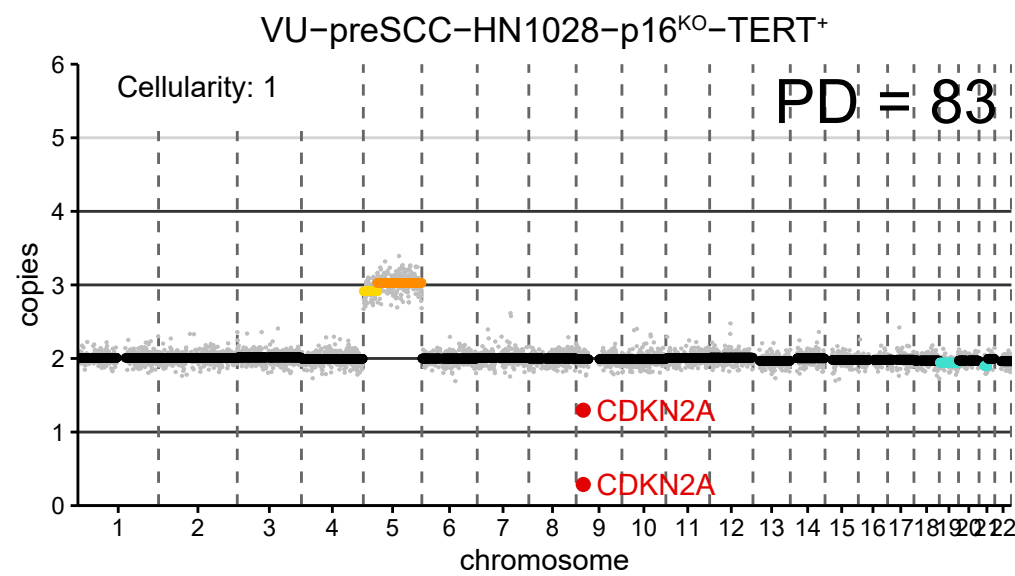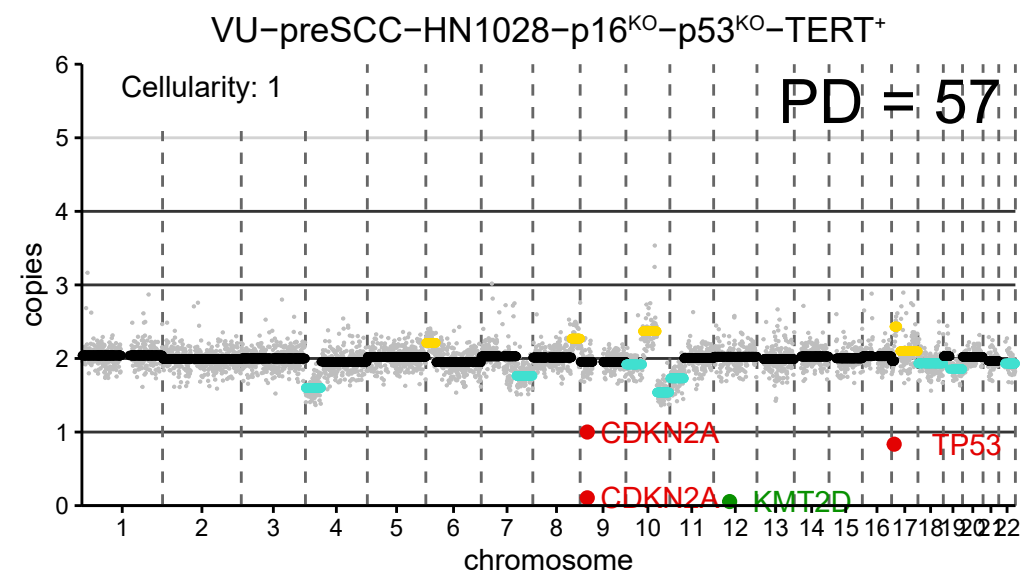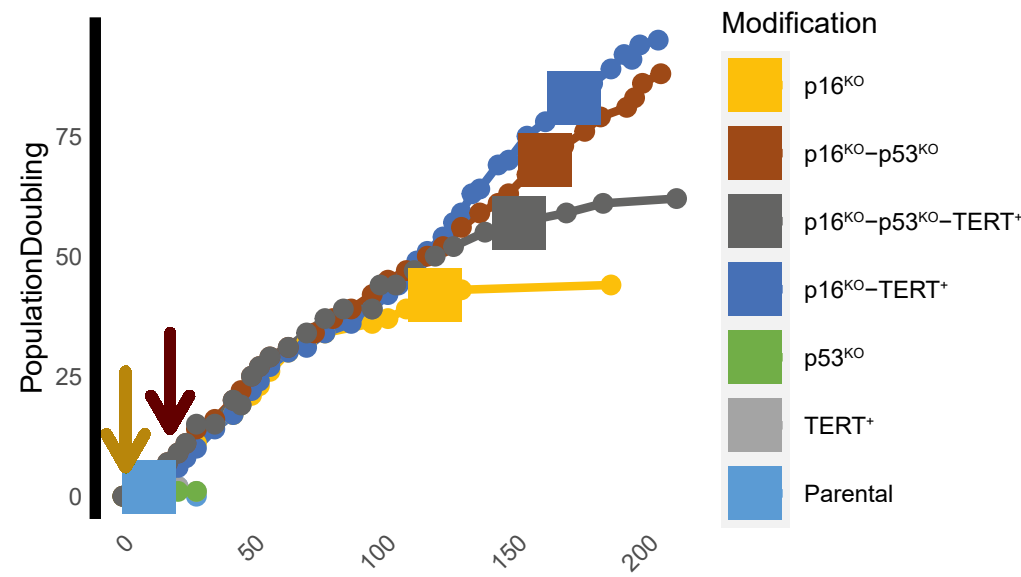

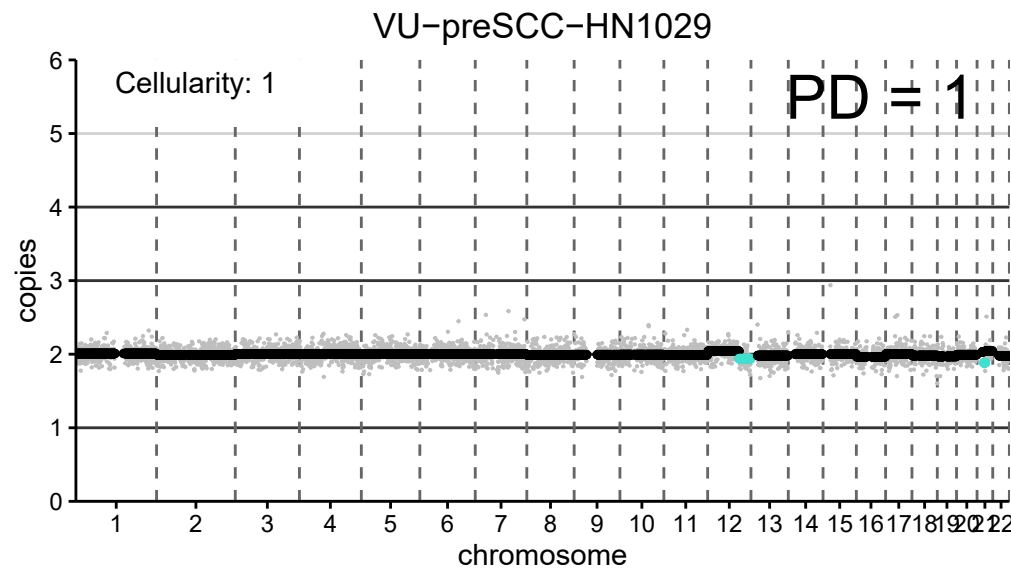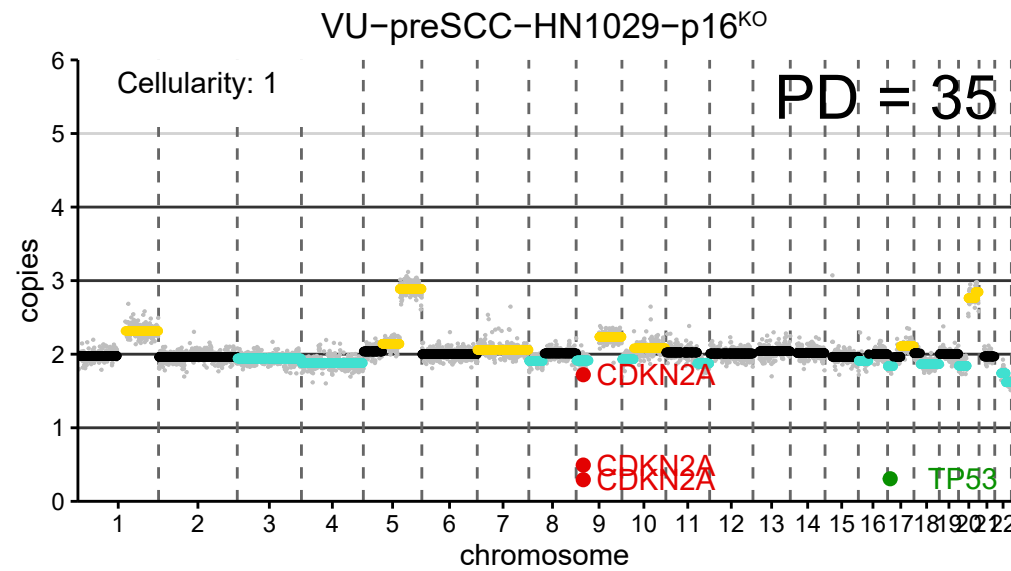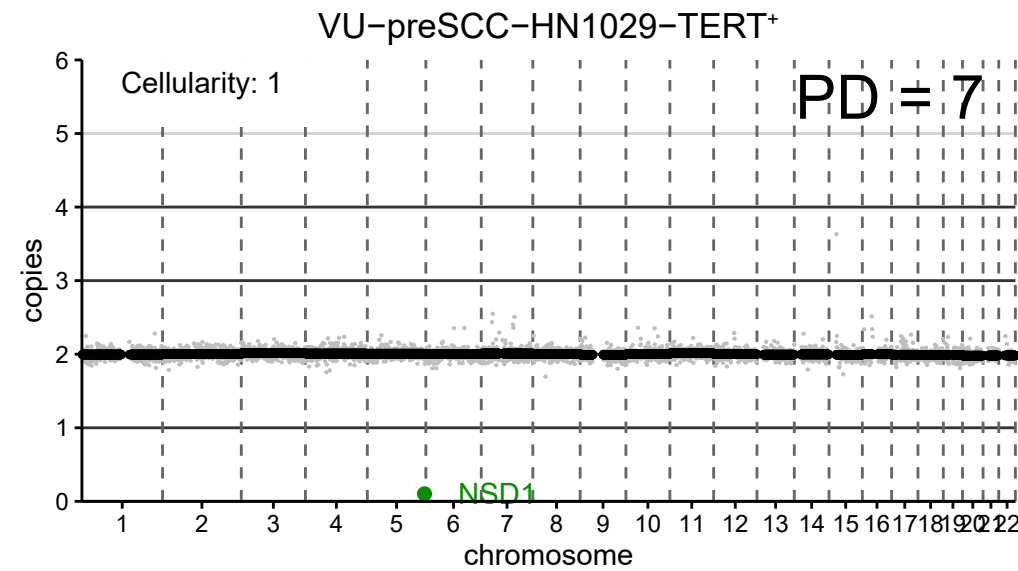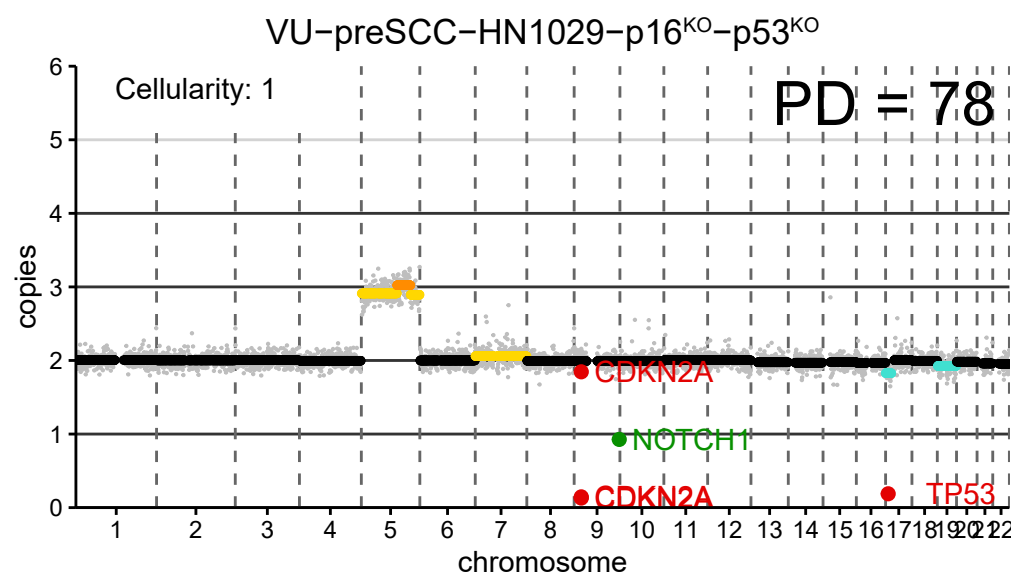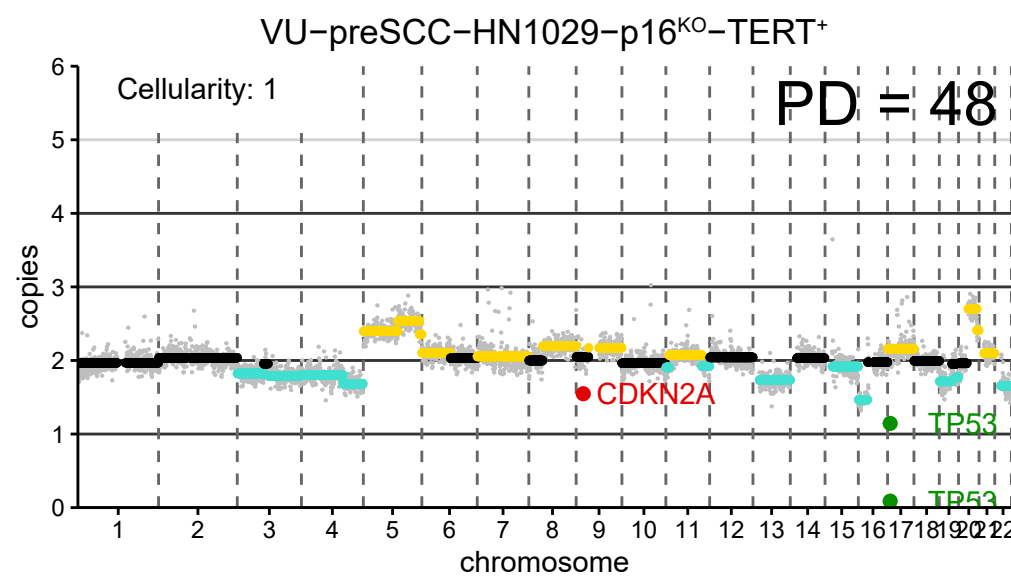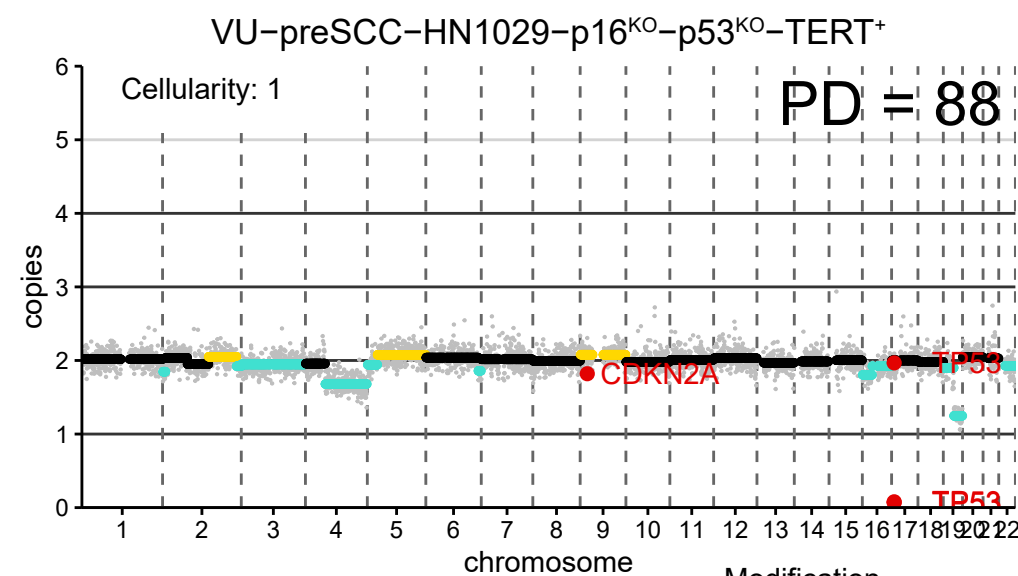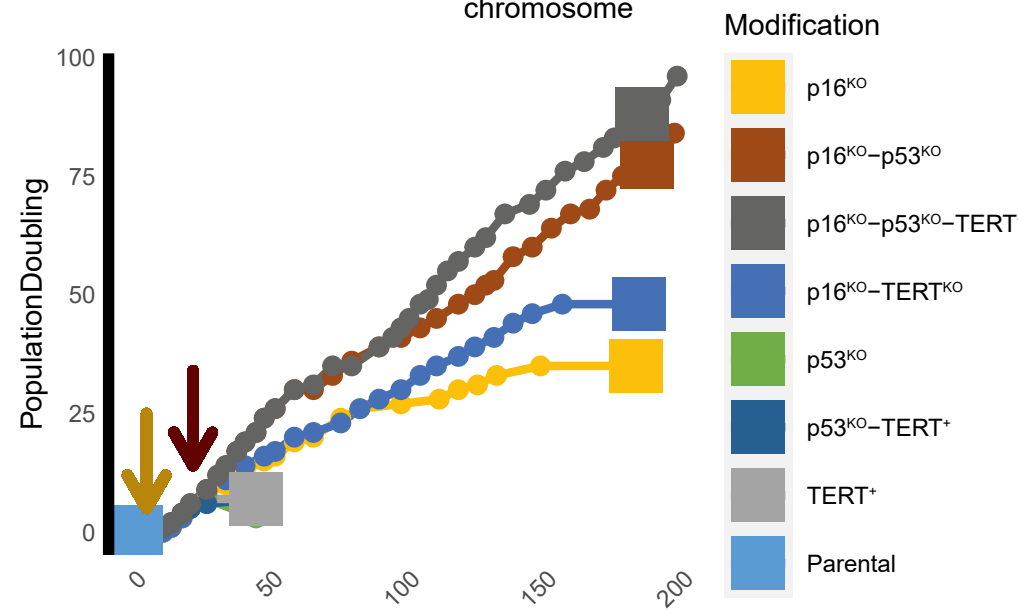

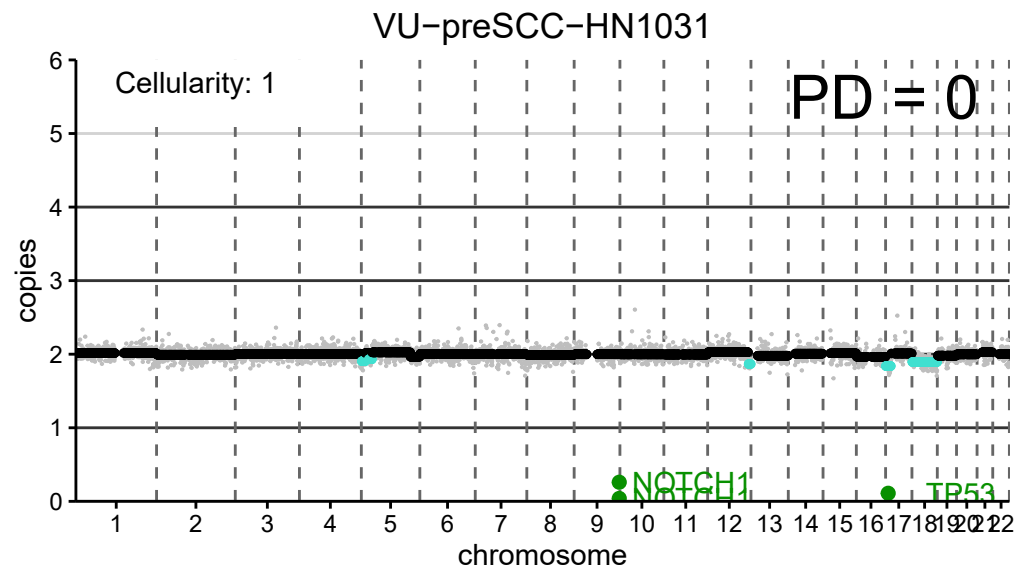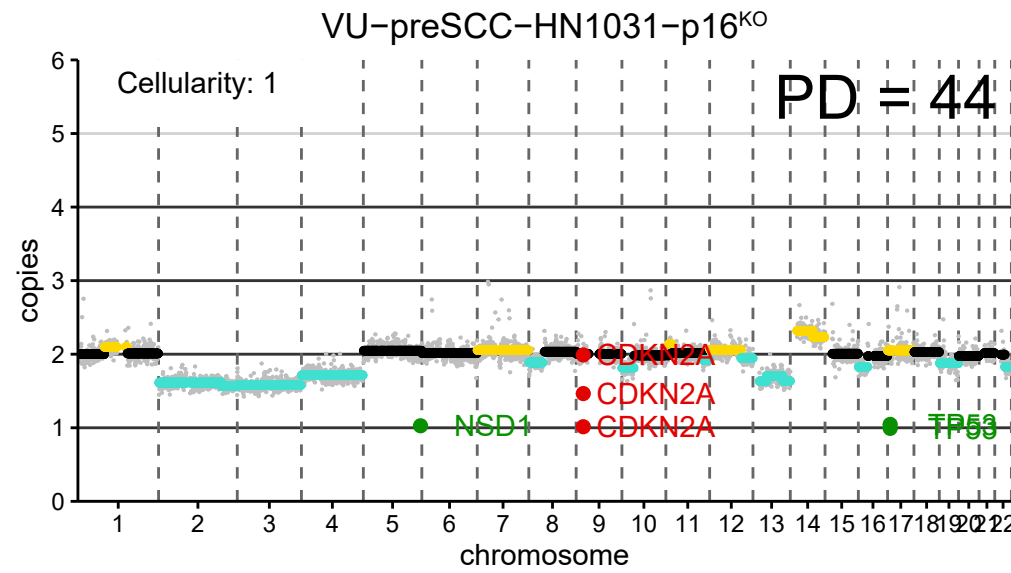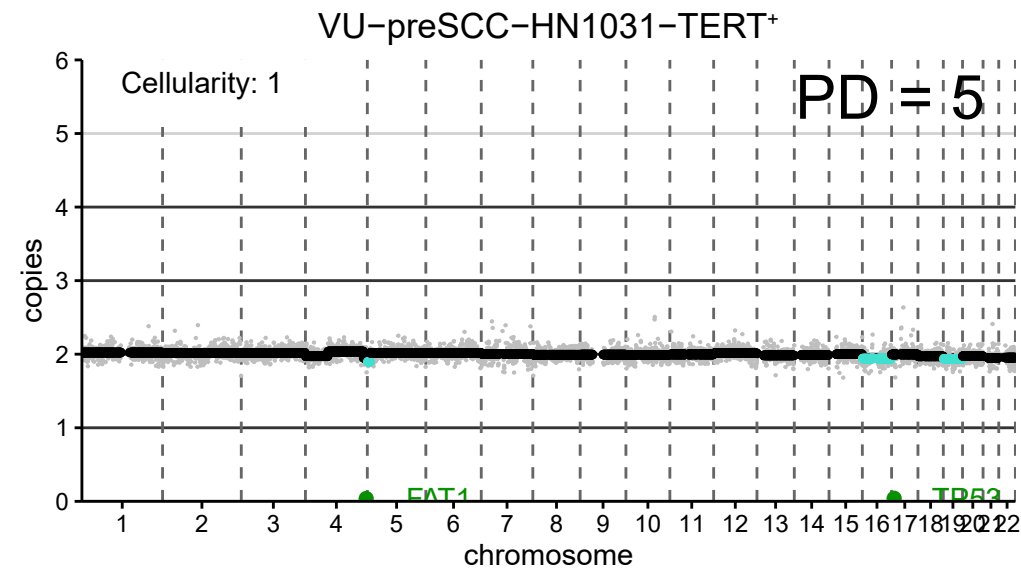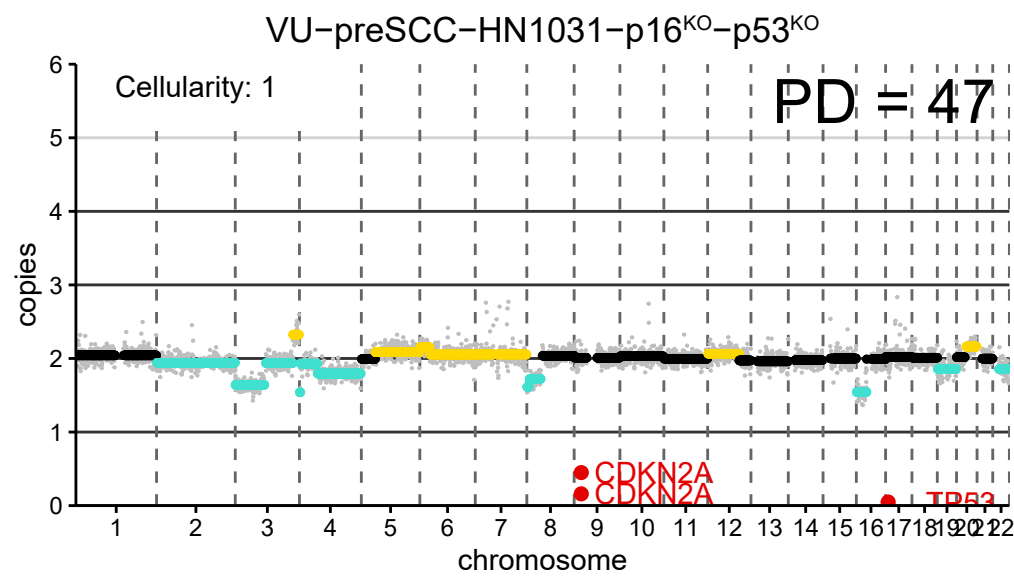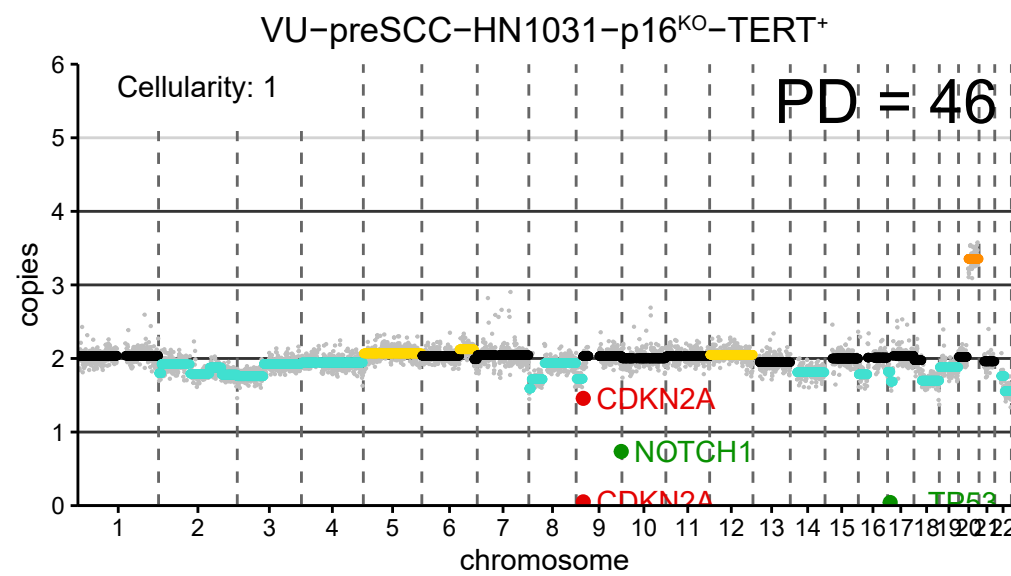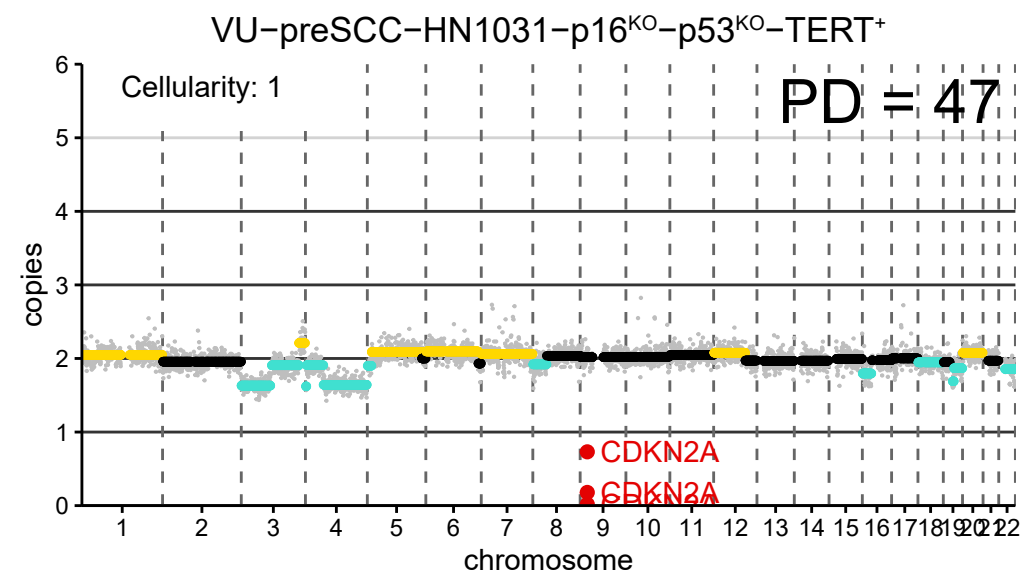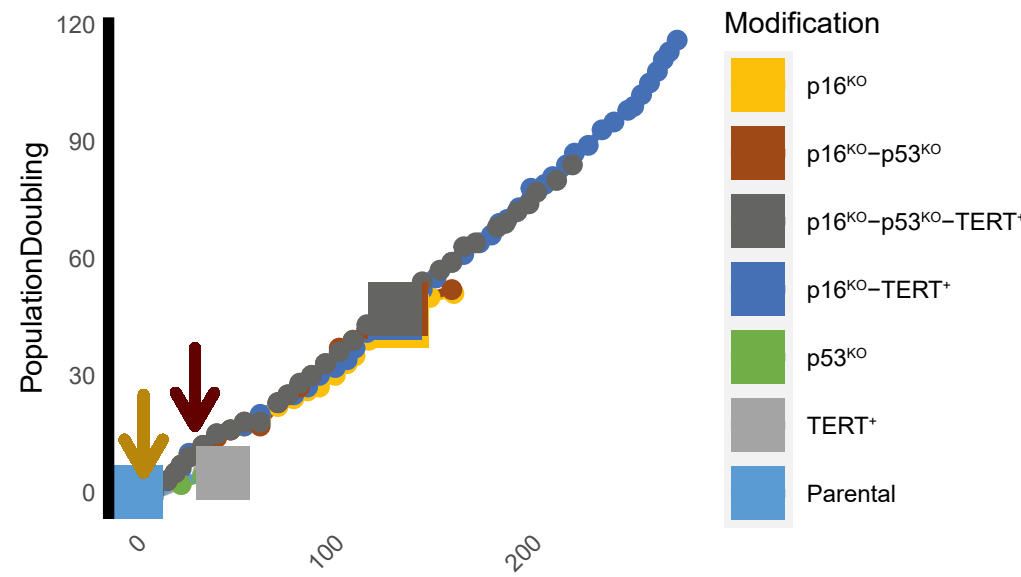

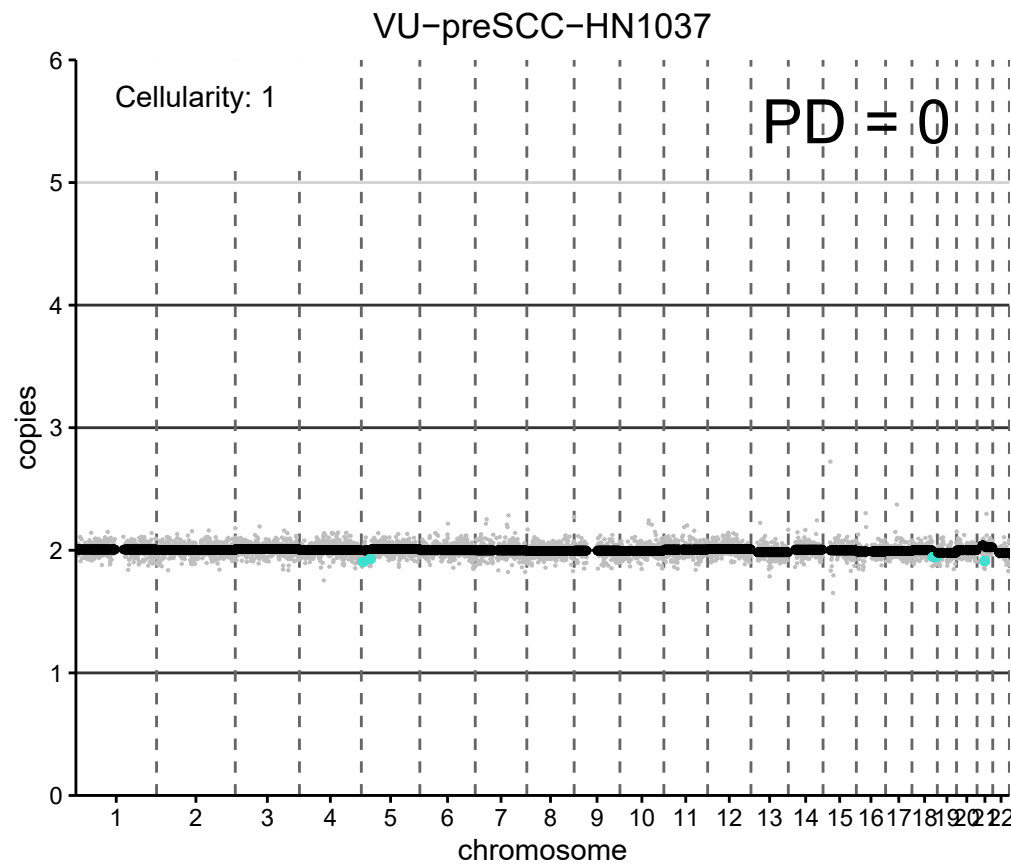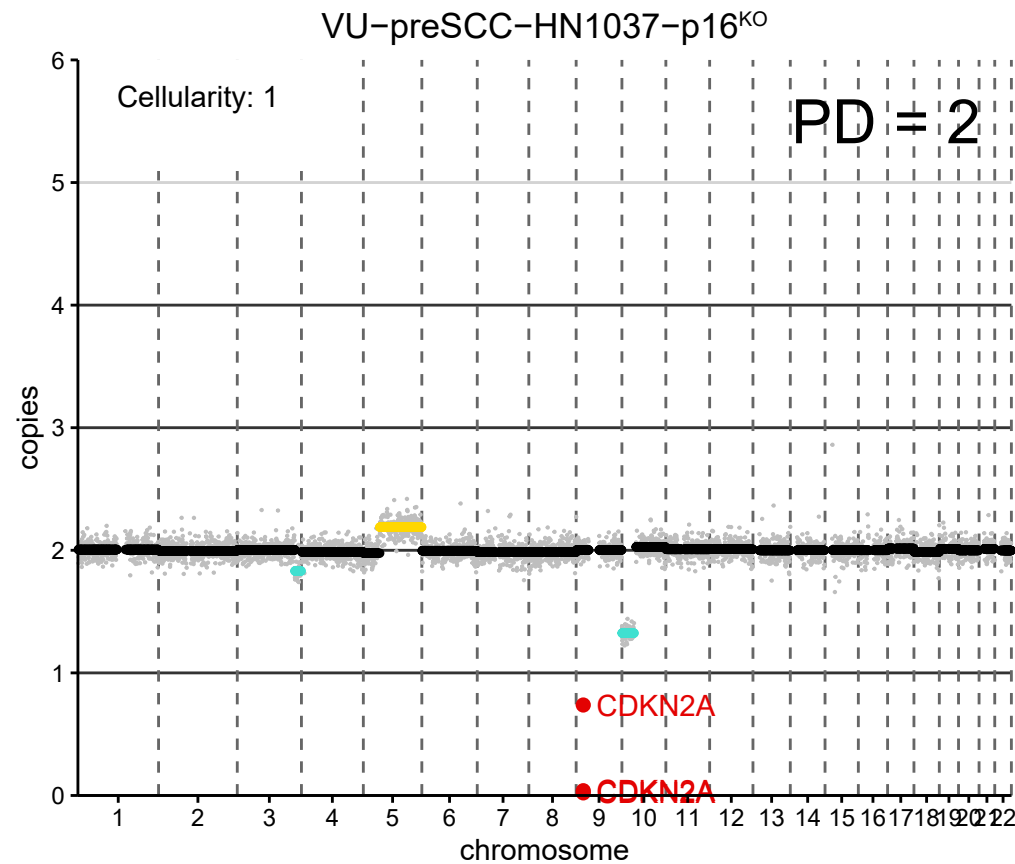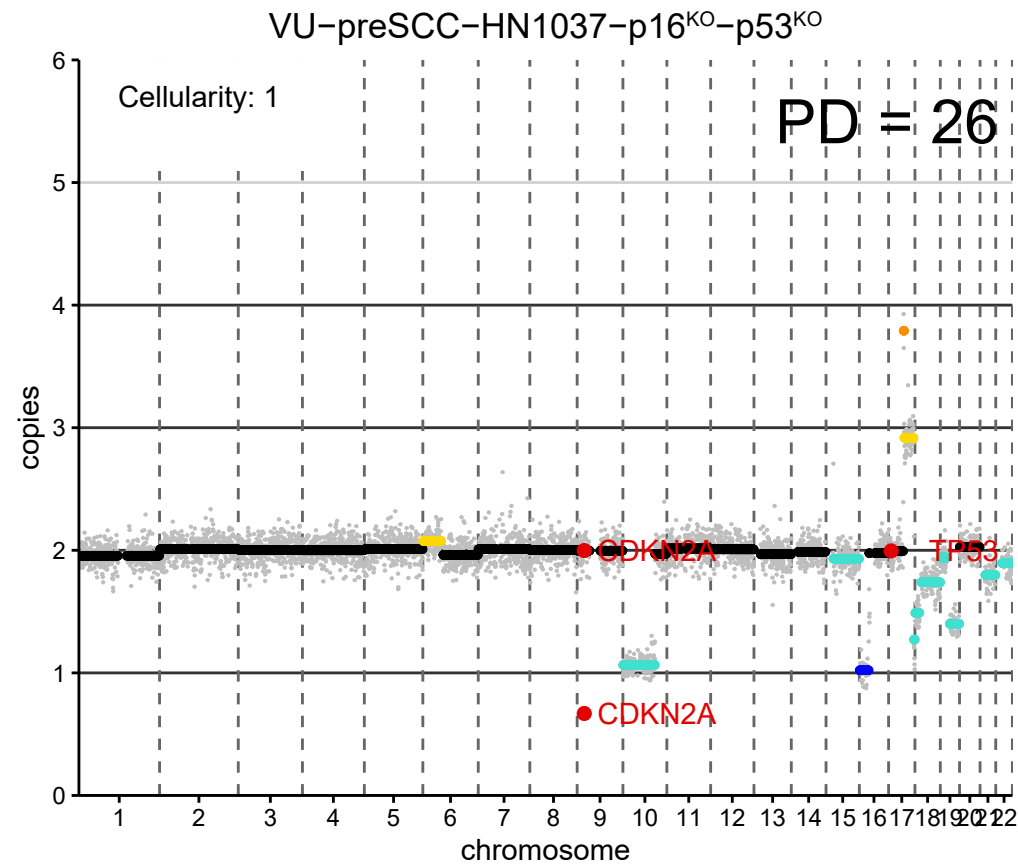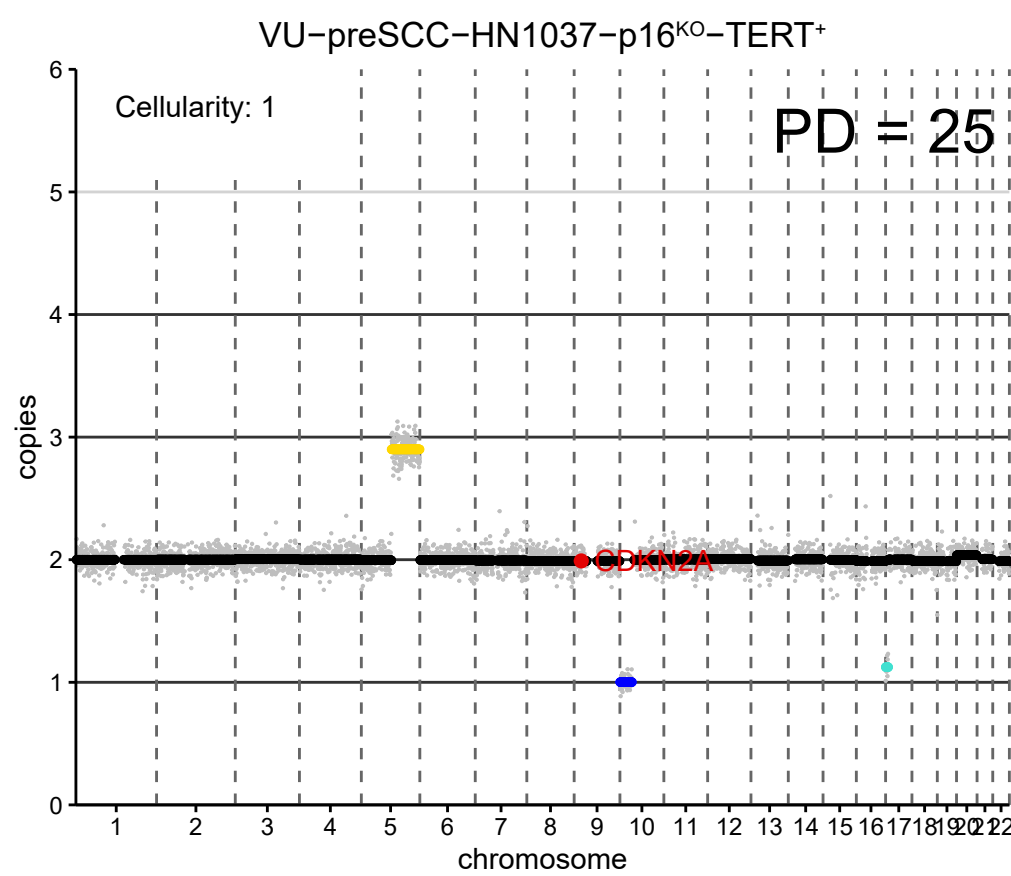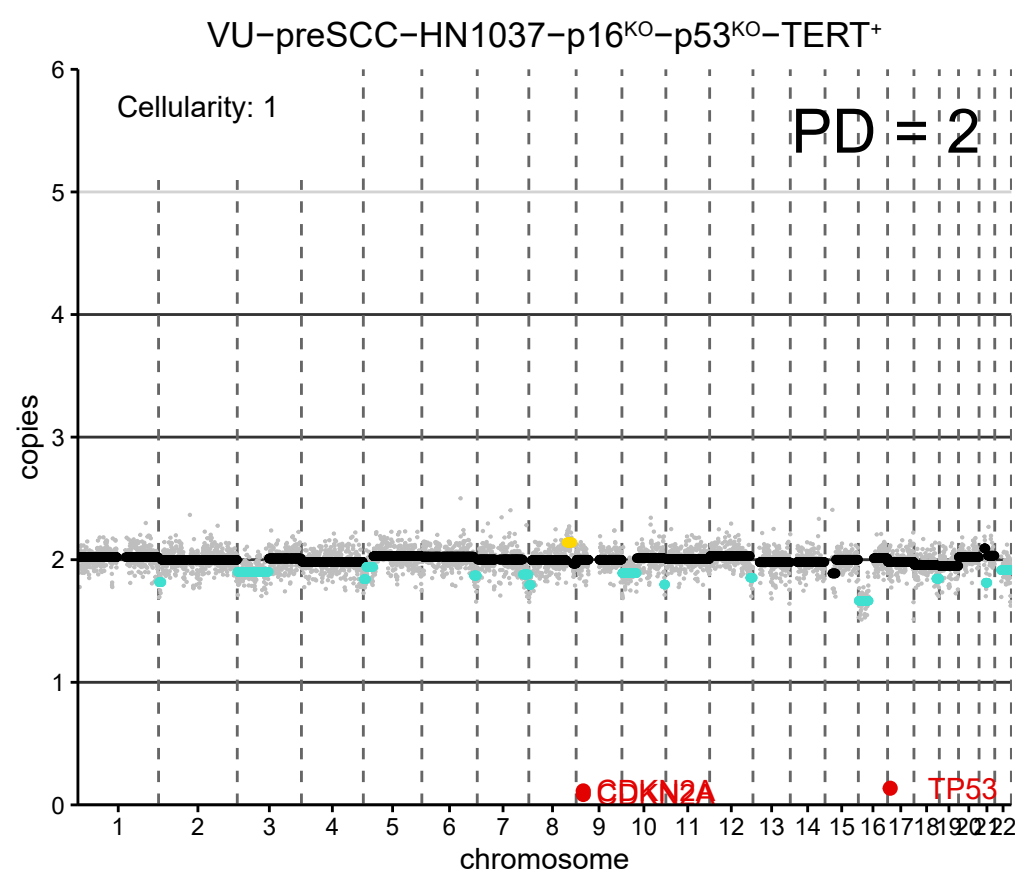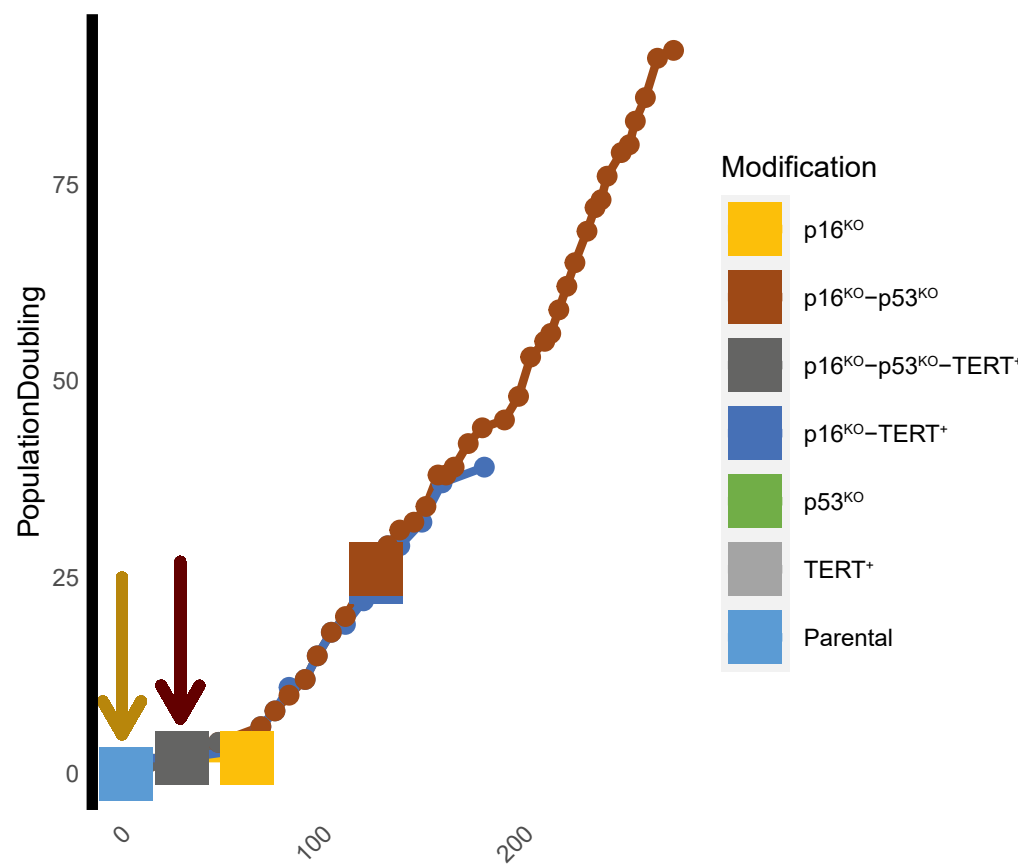

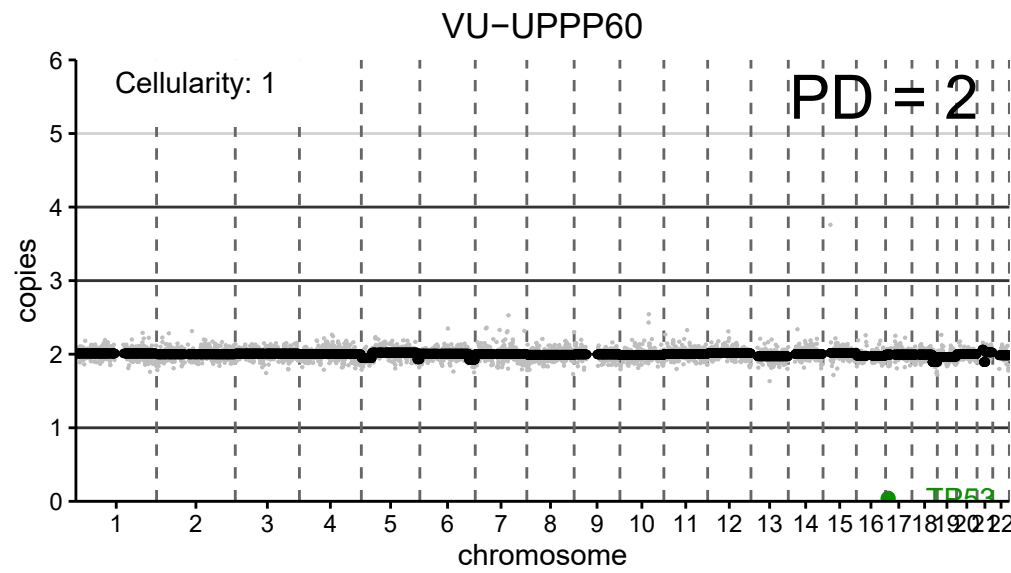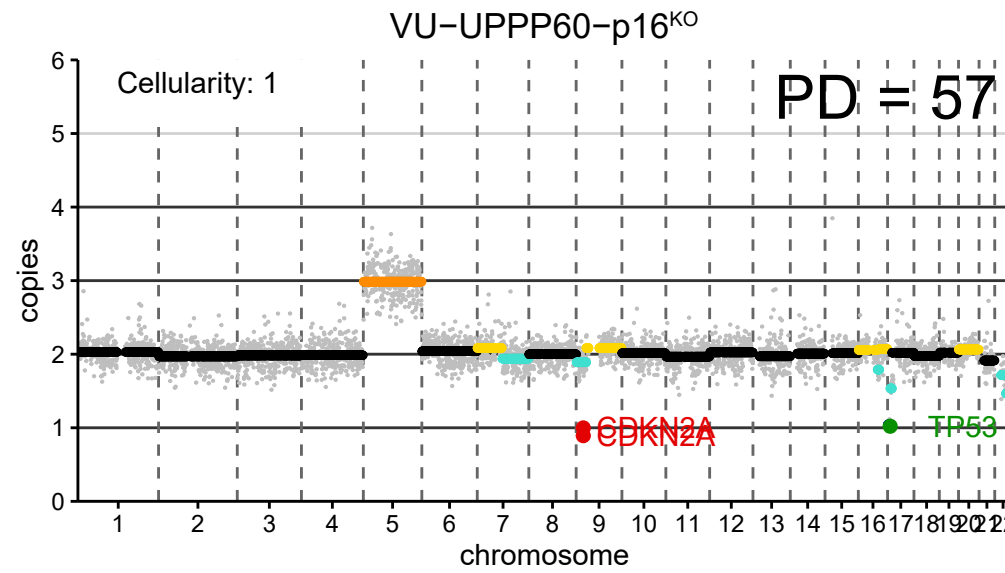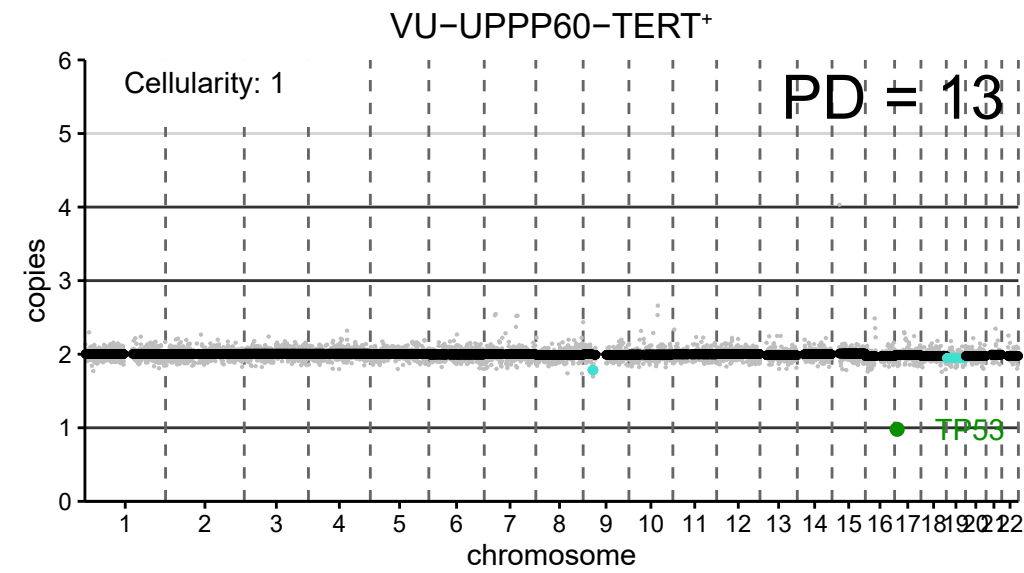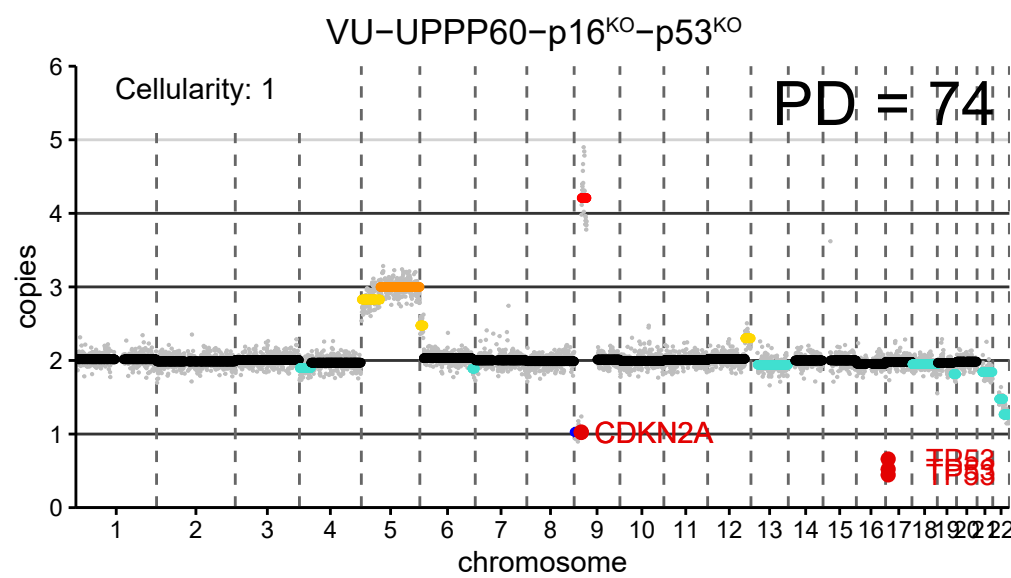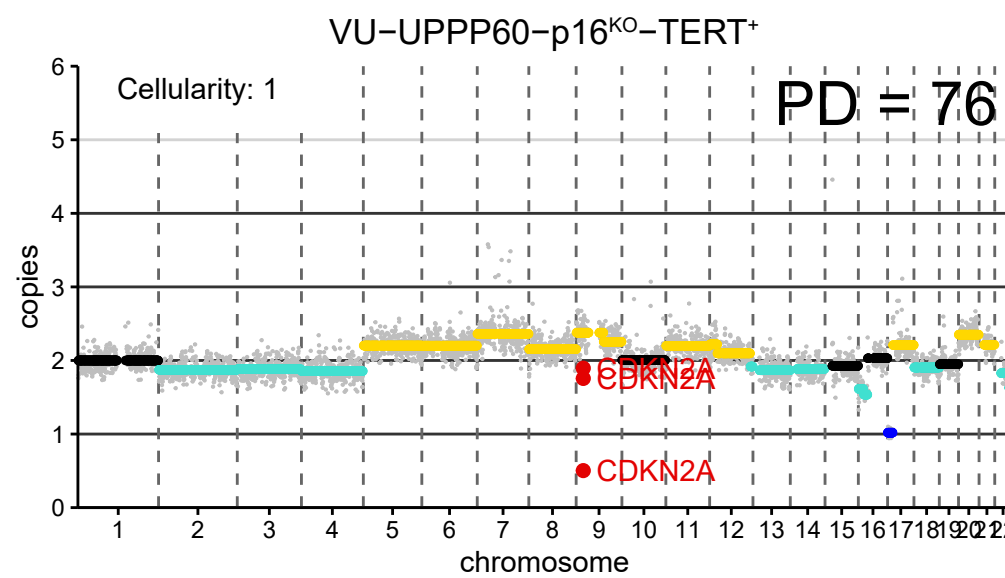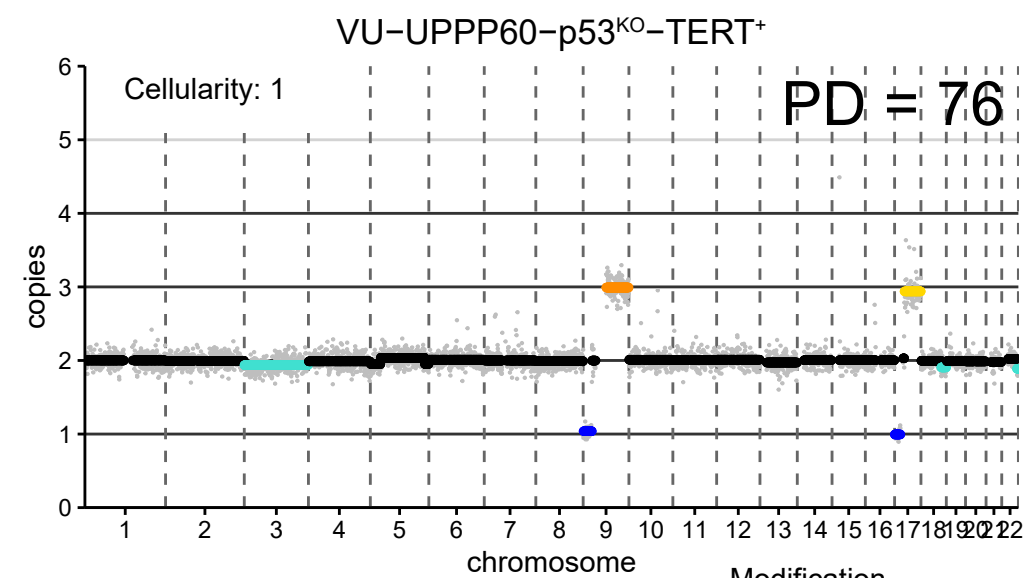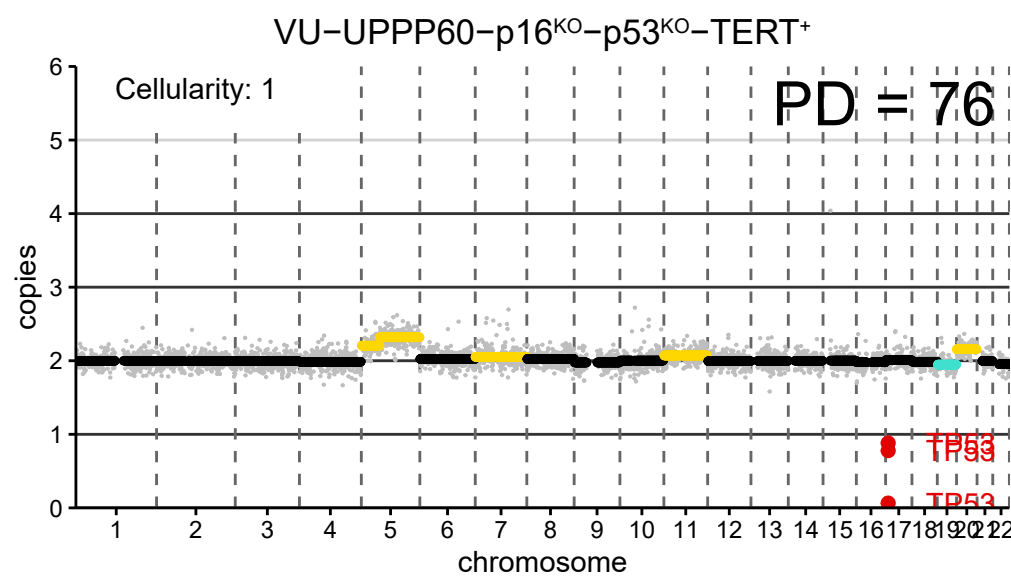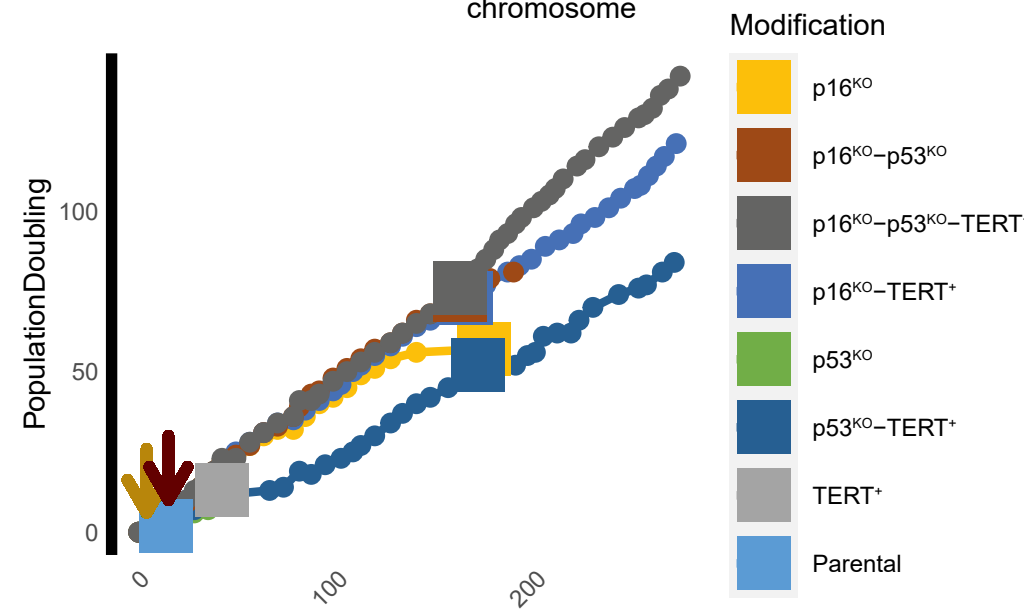

Supplement: Supplementary file 1 [file cells-13-00710-s001.zip › Supplementary_Figure_S1.pdf]

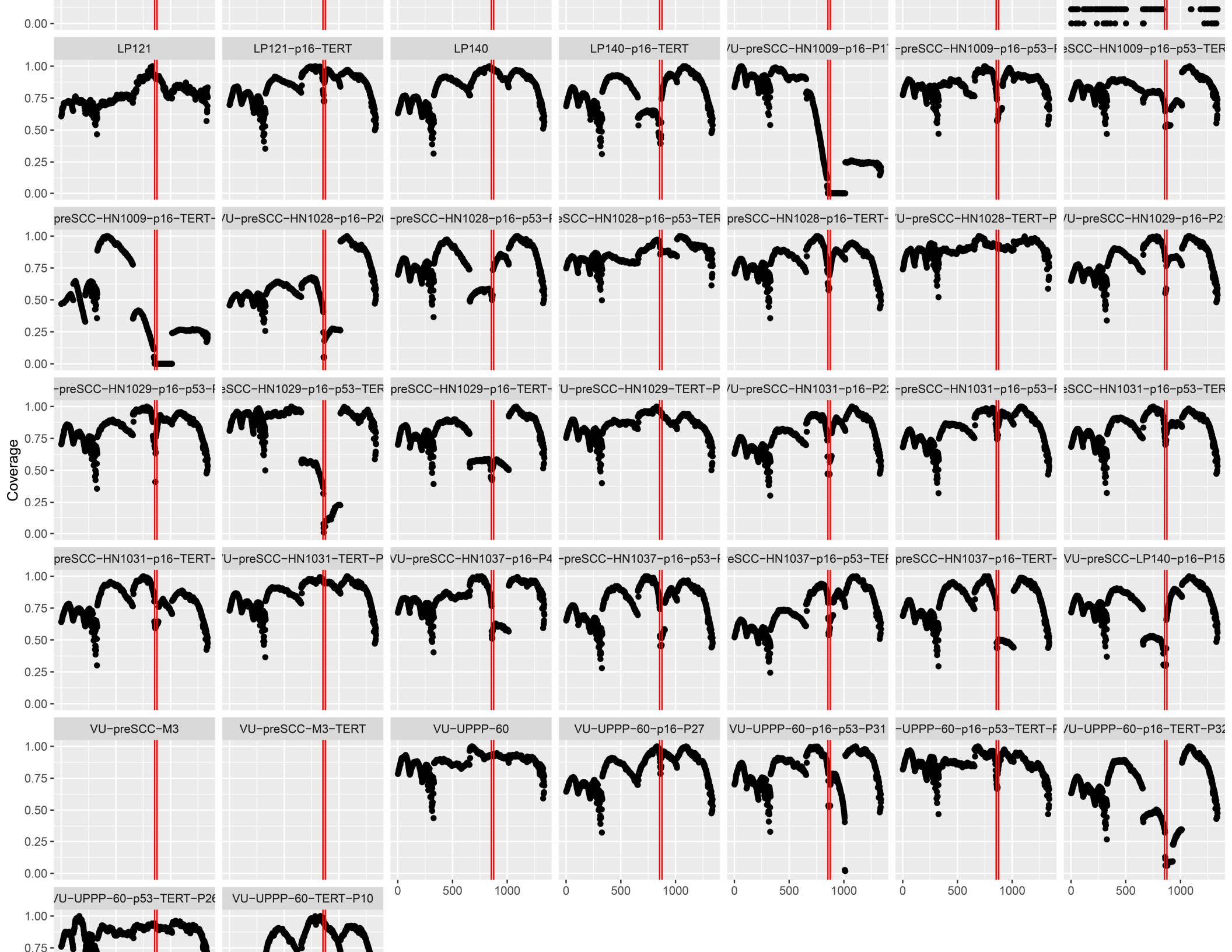

Supplement: Supplementary file 1 [file cells-13-00710-s001.zip › Supplementary_Figure_S2.pdf]

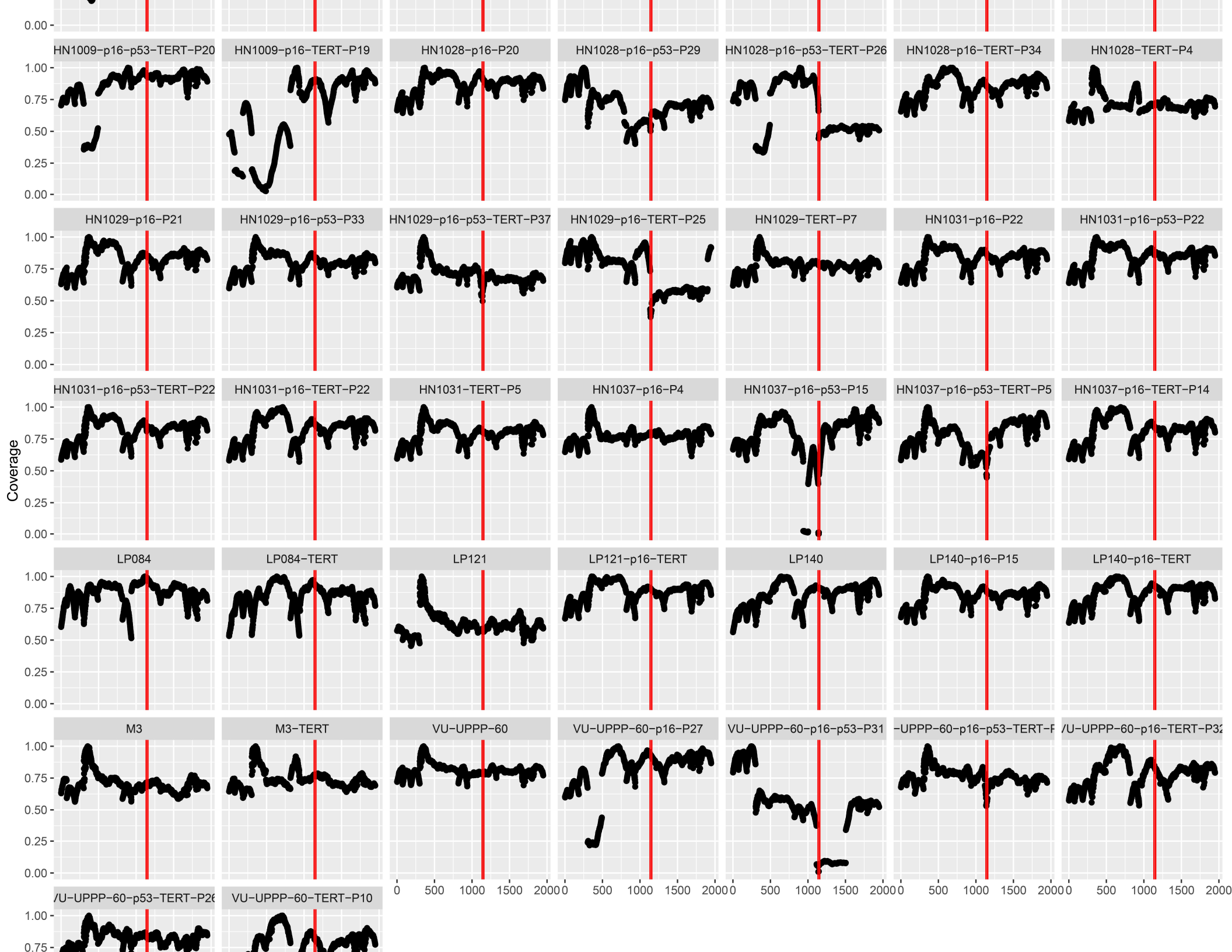

Supplement: Supplementary file 1 [file cells-13-00710-s001.zip › Supplementary_Figure_S3.pdf]

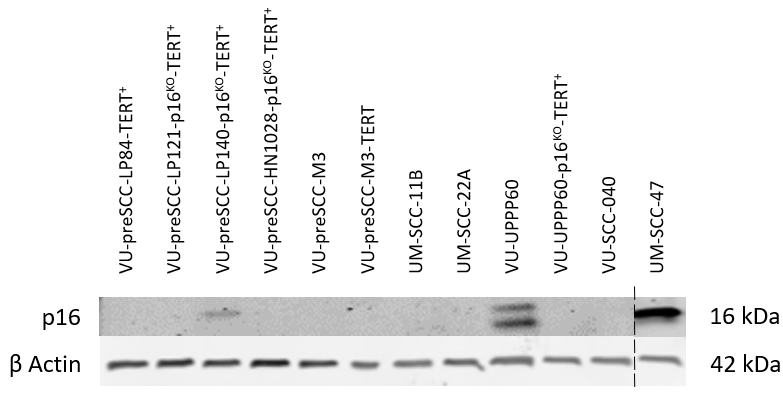

Supplement: Supplementary file 1 [file cells-13-00710-s001.zip › Supplementary_Figure_S4.png]

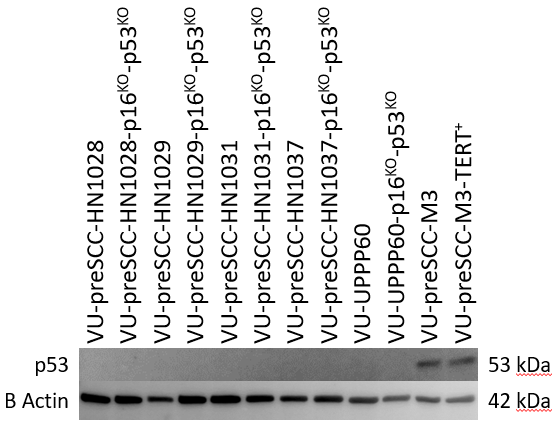

Supplement: Supplementary file 1 [file cells-13-00710-s001.zip › Supplementary_Figure_S5.png]

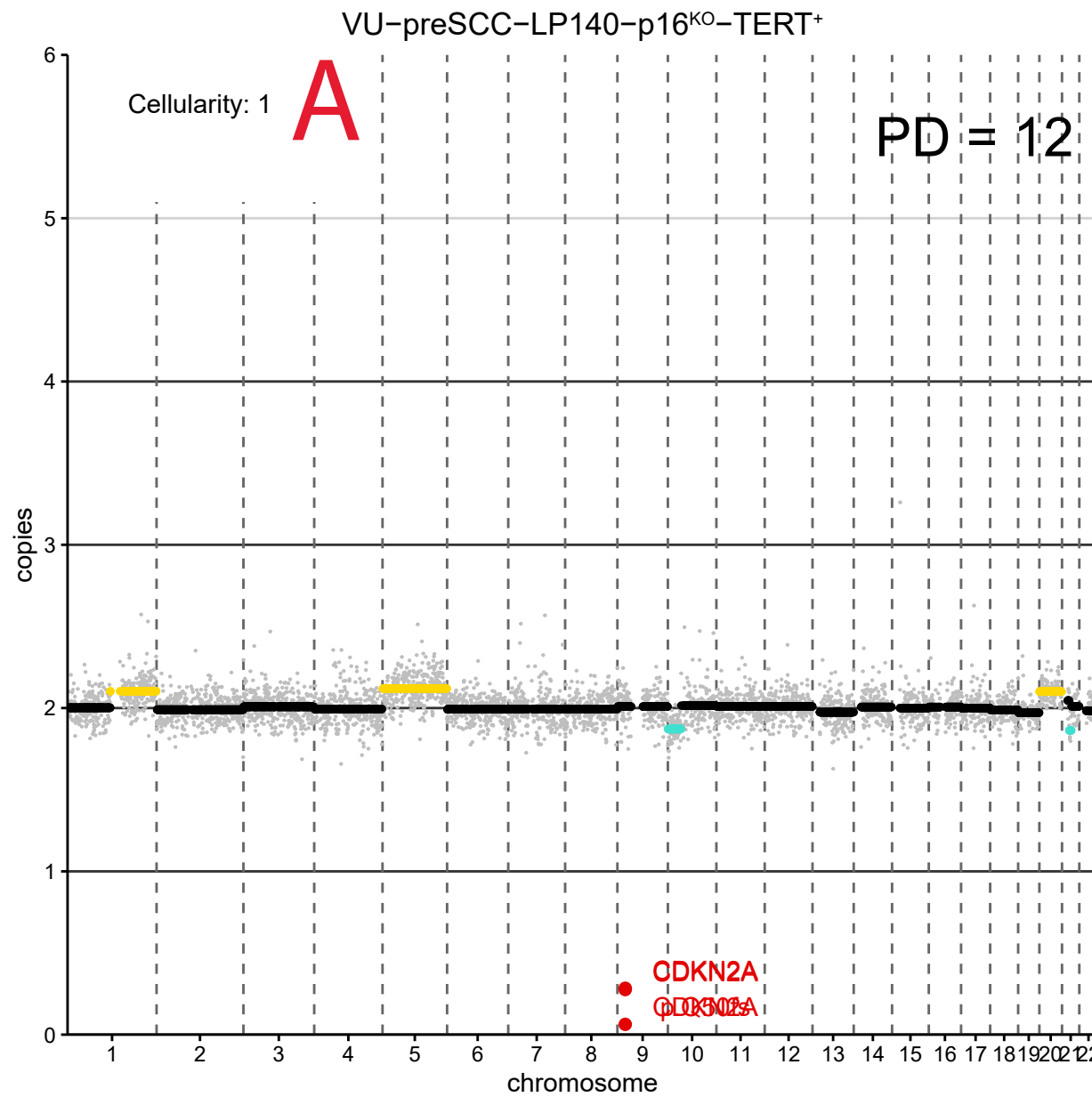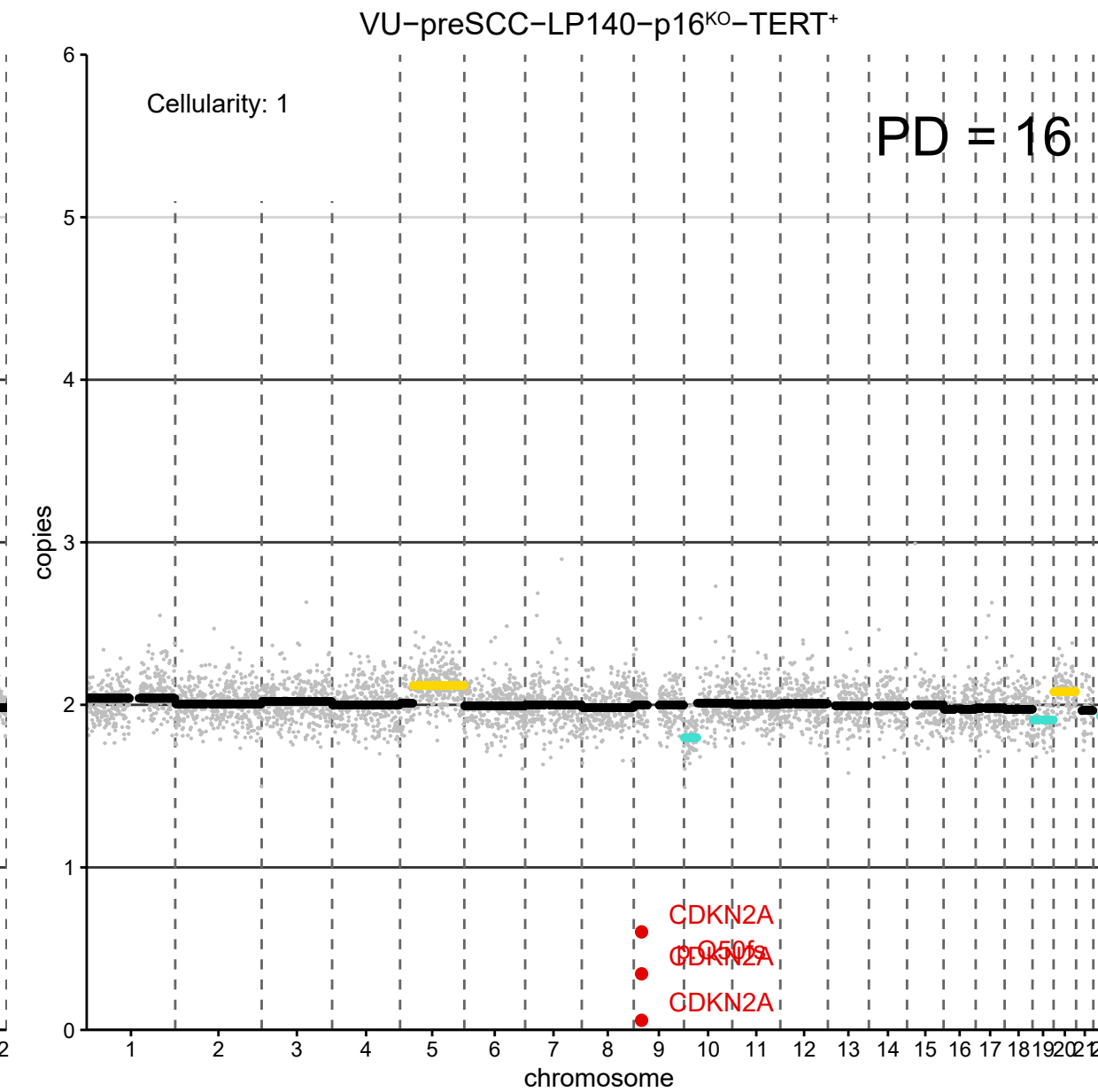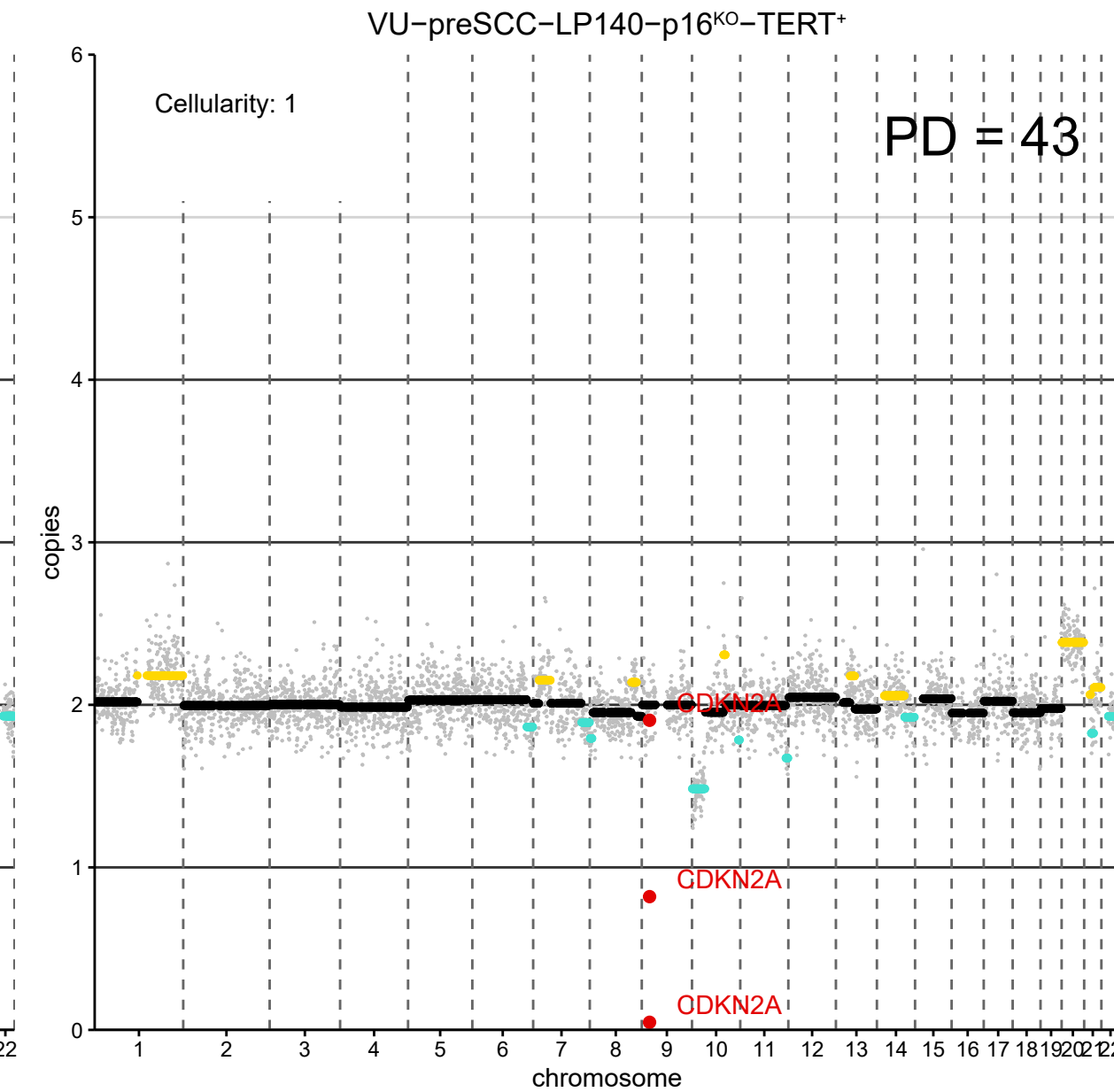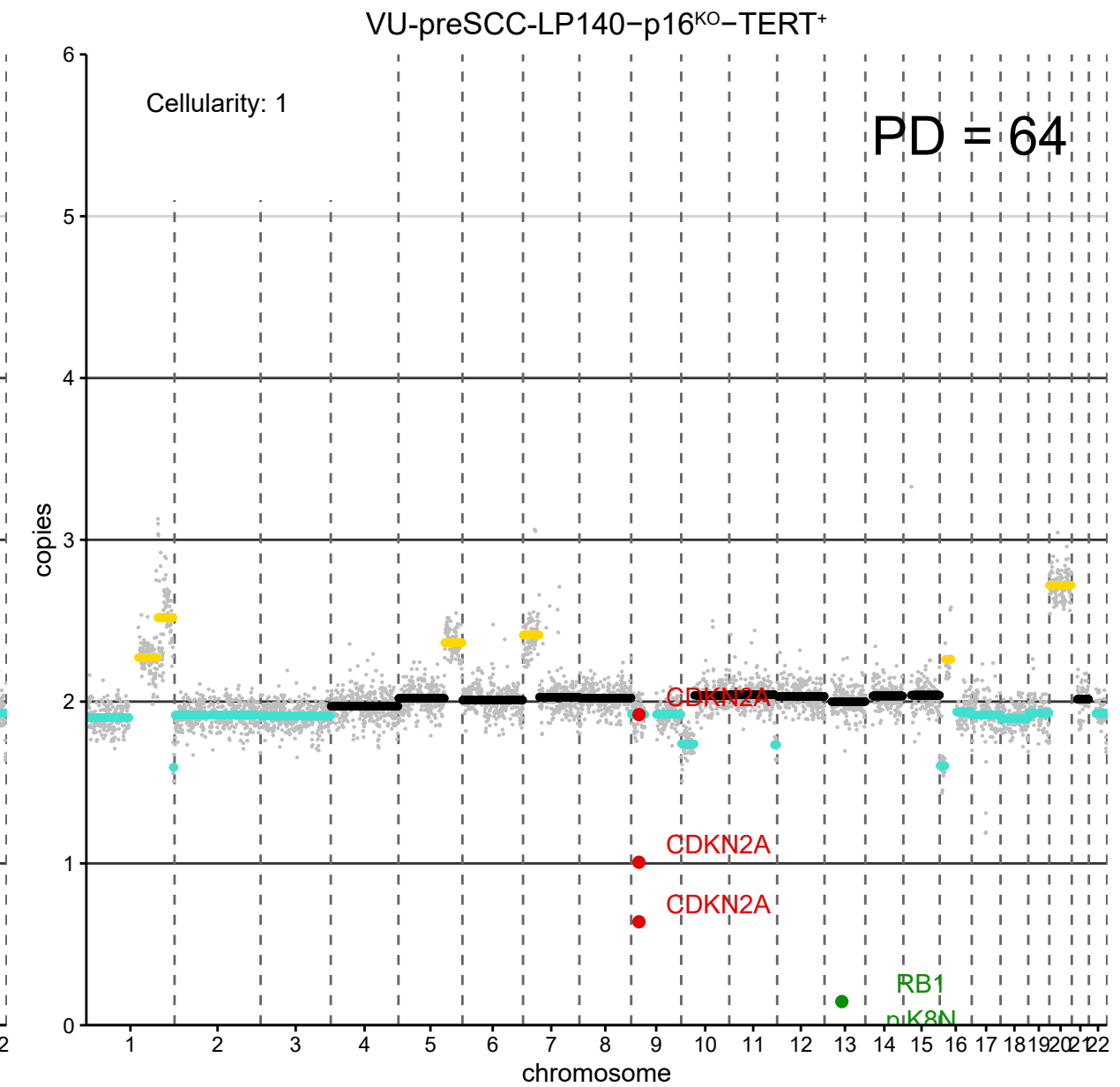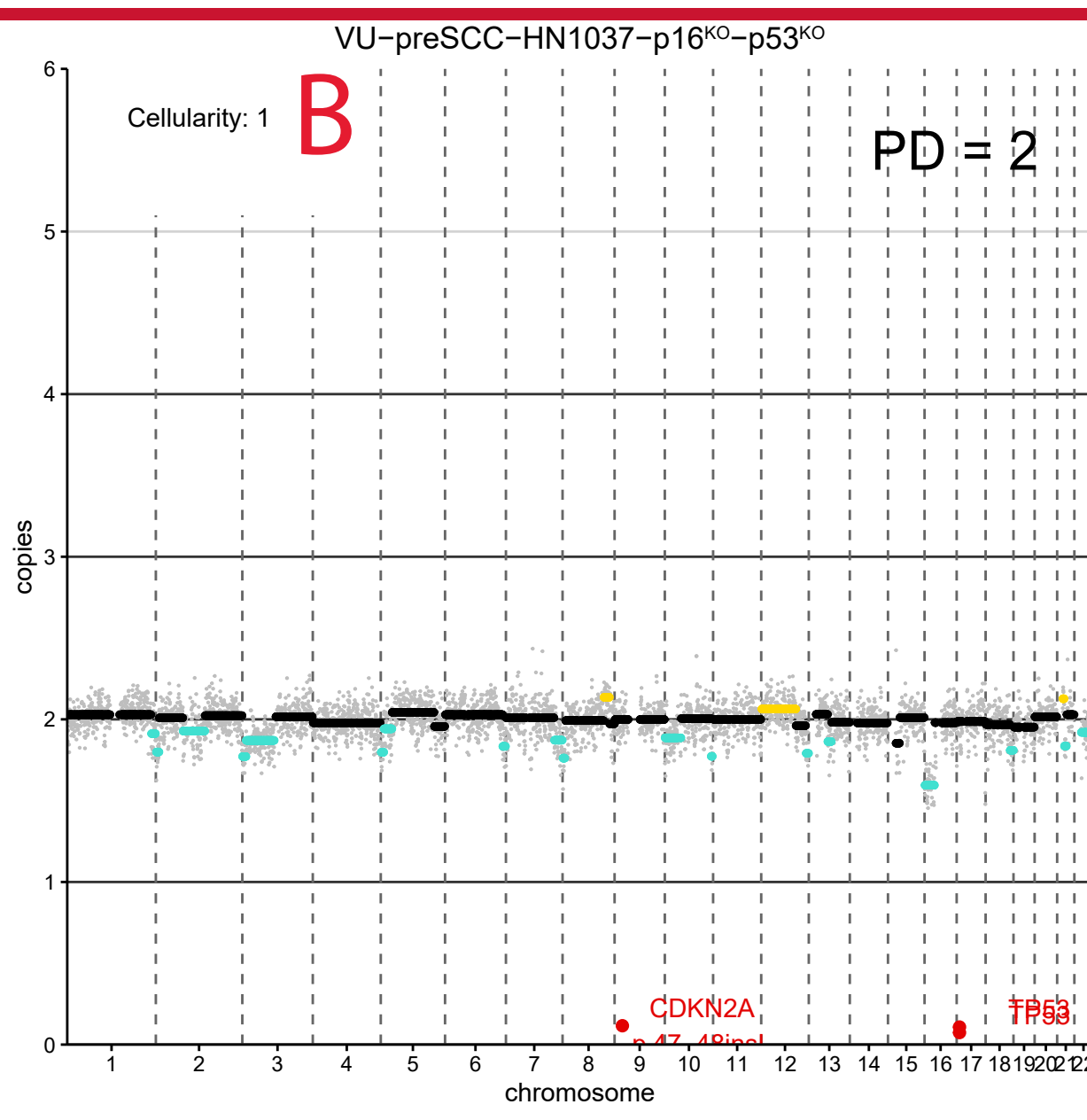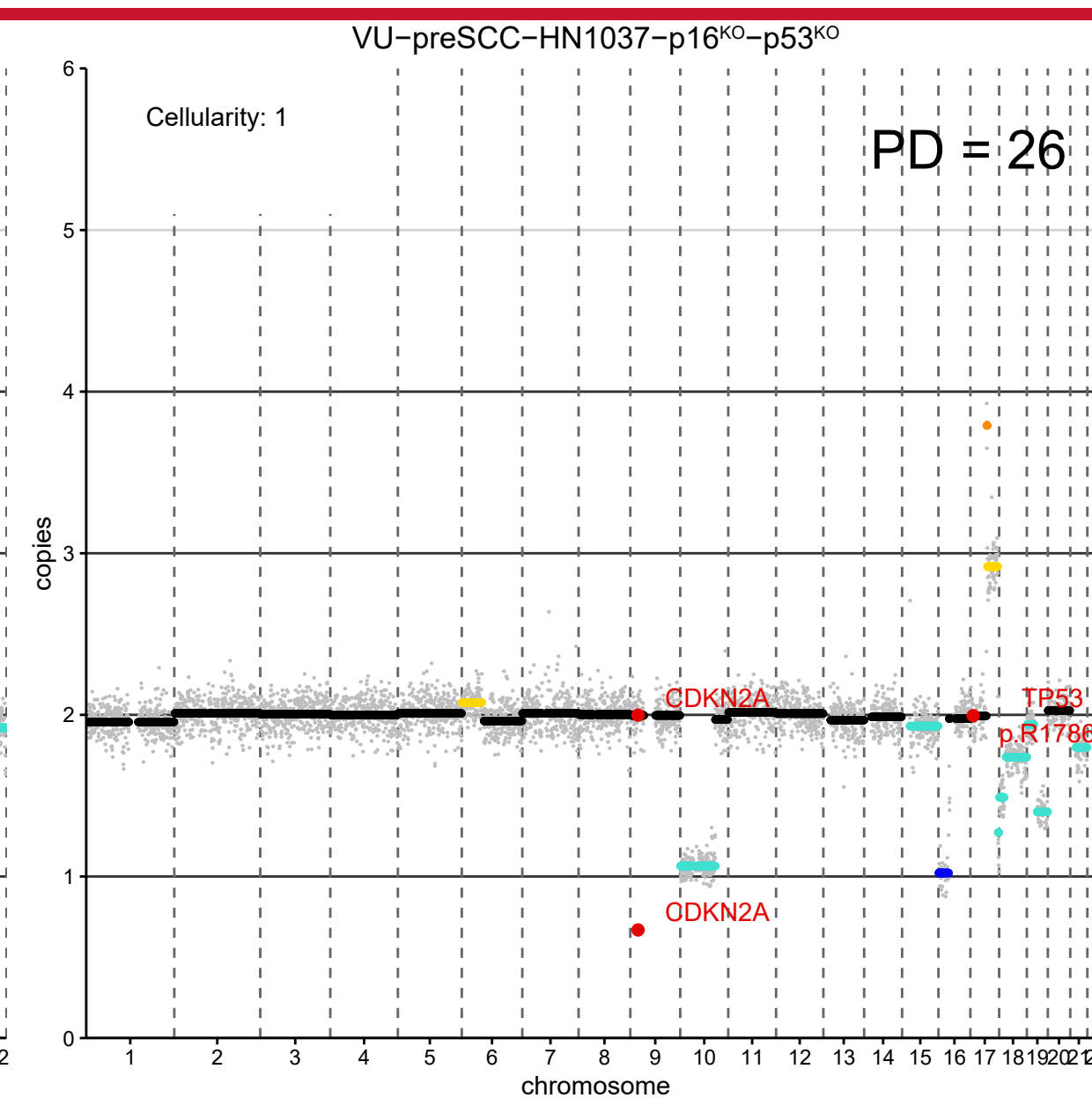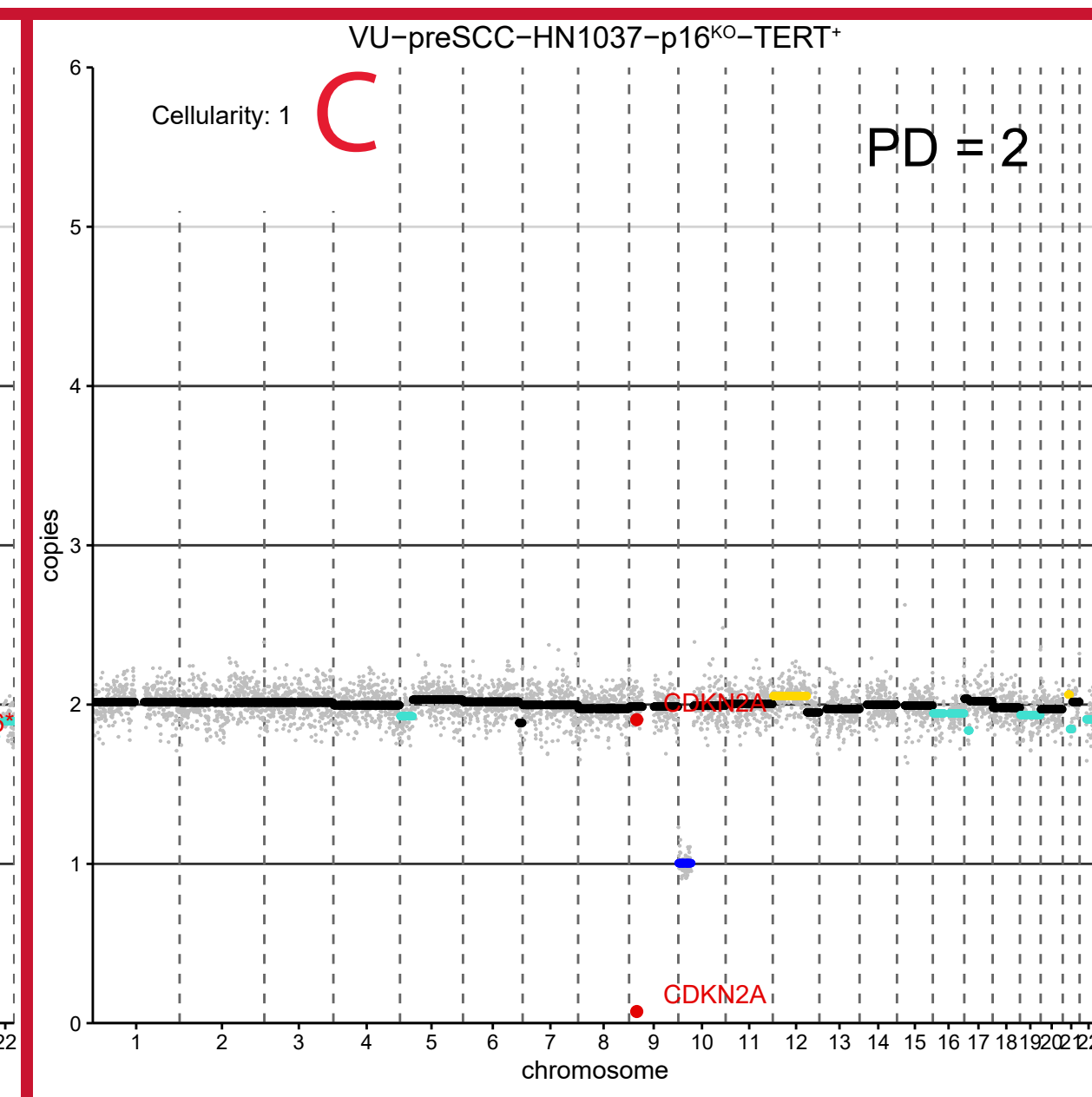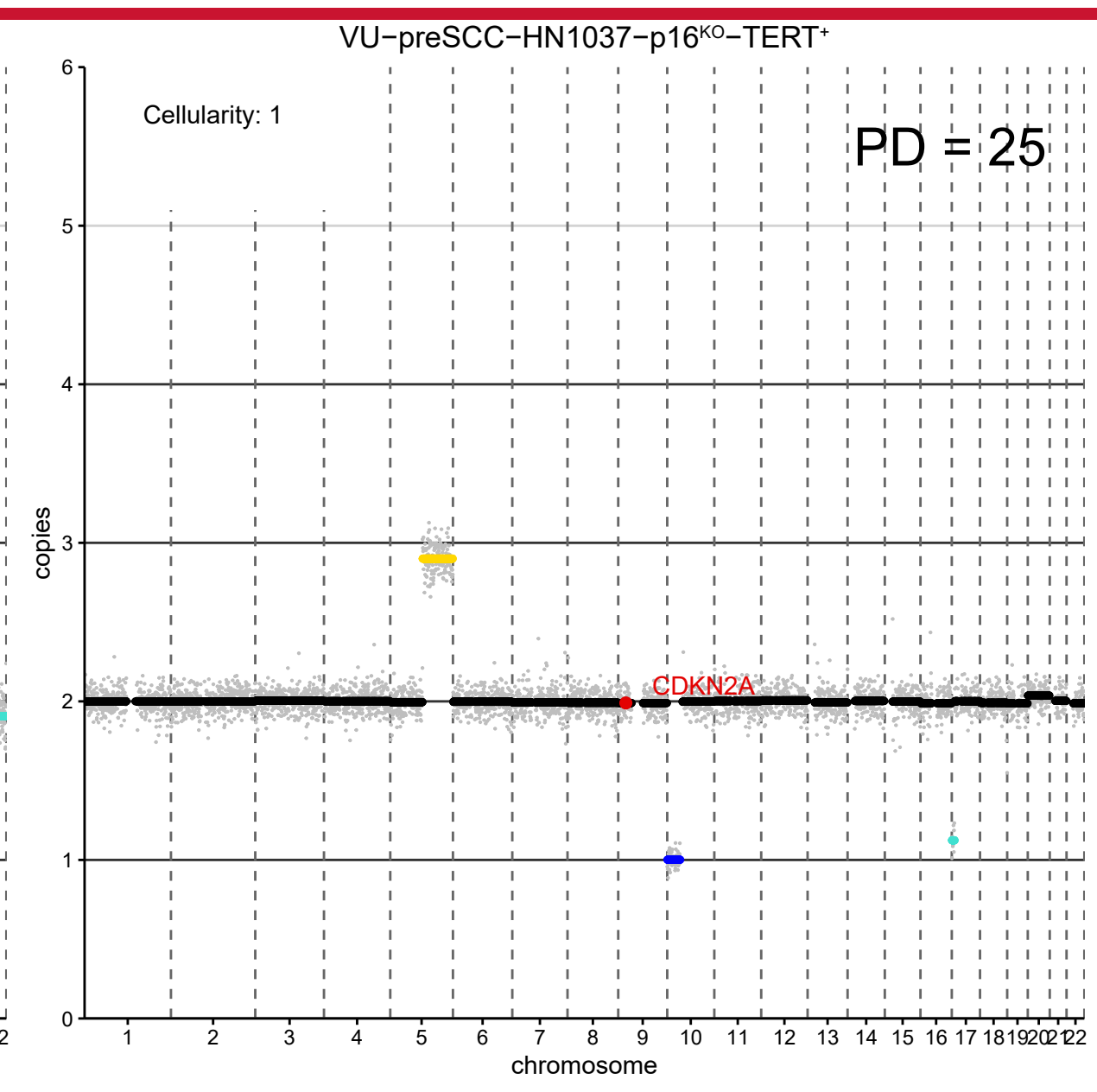

Supplement: Supplementary file 1 [file cells-13-00710-s001.zip › Supplementary_Figure_S7.pdf]
